# Antioxidants of Non-Enzymatic Nature: Their Function in Higher Plant Cells and the Ways of Boosting Their Biosynthesis

**DOI:** 10.3390/antiox12112014

**Published:** 2023-11-17

**Authors:** Natalia N. Rudenko, Daria V. Vetoshkina, Tatiana V. Marenkova, Maria M. Borisova-Mubarakshina

**Affiliations:** 1 Institute of Basic Biological Problems, Federal Research Center “Pushchino Scientific Center for Biological Research of the Russian Academy of Sciences”, Pushchino 142290, Russia; vetoshkinadv@gmail.com (D.V.V.); borisovamm@pbcras.ru (M.M.B.-M.); 2 Federal Research Center Institute of Cytology and Genetics, Siberian Branch of Russian Academy of Sciences, Novosibirsk 630090, Russia; marenkova@bionet.nsc.ru

**Keywords:** higher plants, reactive oxygen species, antioxidants, isoprenoids, plastoquinone, carotenoids, tocopherol, ubiquinone, flavonoids, ascorbic acid, glutathione, transgenesis, CRISPR/Cas9

## Abstract

Plants are exposed to a variety of abiotic and biotic stresses leading to increased formation of reactive oxygen species (ROS) in plant cells. ROS are capable of oxidizing proteins, pigments, lipids, nucleic acids, and other cell molecules, disrupting their functional activity. During the process of evolution, numerous antioxidant systems were formed in plants, including antioxidant enzymes and low molecular weight non-enzymatic antioxidants. Antioxidant systems perform neutralization of ROS and therefore prevent oxidative damage of cell components. In the present review, we focus on the biosynthesis of non-enzymatic antioxidants in higher plants cells such as ascorbic acid (vitamin C), glutathione, flavonoids, isoprenoids, carotenoids, tocopherol (vitamin E), ubiquinone, and plastoquinone. Their functioning and their reactivity with respect to individual ROS will be described. This review is also devoted to the modern genetic engineering methods, which are widely used to change the quantitative and qualitative content of the non-enzymatic antioxidants in cultivated plants. These methods allow various plant lines with given properties to be obtained in a rather short time. The most successful approaches for plant transgenesis and plant genome editing for the enhancement of biosynthesis and the content of these antioxidants are discussed.

## 1. Introduction

In all aerobic organisms, the interaction between molecular oxygen and various cellular components invariably gives rise to the production of reactive oxygen species (ROS). In higher plants, these components primarily include the carriers of the electron transport chains in both photosynthetic and respiratory apparatuses and of the short plasma membrane chain. Even under optimal operating conditions, ROS are formed at a low level [1]. Stress conditions amplify ROS production in different cellular compartments, such as chloroplasts [2,3,4], peroxisomes [5], mitochondria [6], and the plasma membrane [7]. The physiological functions of ROS largely depend on their chemical properties, their formation site, and the concentration of the ROS. This concentration is influenced by the rate of ROS formation and the rate of their neutralization by the antioxidant systems.

The antioxidant systems in higher plants are represented by antioxidant enzymes and non-enzymatic antioxidants. Antioxidant enzymes exist in multiple molecular forms (isoenzymes) across different cell organelles, mainly within their aqueous phases (for review, see [8]). For example, superoxide dismutases (SODs), which detoxify superoxide anion radicals, can be classified as CuZnSOD, MnSOD, or FeSOD, based on the metal cofactor. These isoenzymes are localized in distinct cell compartments: in chloroplasts (for FeSOD and CuZnSOD), cytosol (for CuZnSOD), and mitochondria (for MnSOD). Catalase, ascorbate peroxidases (APXs), Cys peroxiredoxins, and various other peroxidases are the most abundant antioxidant enzymes, which catalyze the reduction of hydrogen peroxide (H_2_O_2_) to water. APXs (thylakoid-bound APX and stromal APX) and Cys peroxiredoxins detoxify H_2_O_2_ in chloroplasts and cytoplasmic and peroxisomal APX in cytoplasm and peroxisomes, respectively. Catalase functions in glyoxysomes and peroxisomes. 

Representatives of non-enzymatic antioxidants in higher plants are ascorbic acid (vitamin C), glutathione, flavonoids, isoprenoids, carotenoids, tocopherol (vitamin E), ubiquinone, and plastoquinone. Similar to antioxidant enzymes, non-enzymatic antioxidants function in different cell compartments, but in both aqueous (ascorbic acid, glutathione, and flavonoids) and membrane phases (flavonoids and isoprenoids). Recently, the potential antioxidant activity of compounds that were not previously considered as such (sugars, proline, etc.) has been actively studied. However, they are not the subject of this review, as the mechanism of their antioxidant activity remains unclear and may be associated with their protective and signaling effects.

Over the last few decades, scientists not only successfully increased the intensity of biosynthesis of antioxidant enzymes, but also multiplied the level of the antioxidants to improve plants sustainability. Although creating mutant plants with modified biosynthesis of non-enzymatic antioxidants is complicated, since multiple pathways and enzymes are involved in their biosynthesis, these plants may have some advantages over plants with overexpression of antioxidant enzymes. First of all, low molecular weight antioxidants can interact with different types of ROS, not just one, as in the case of antioxidant enzymes. Moreover, it is known that stress conditions often result in exhaustion of the substrates of the antioxidant enzymes, especially at the initial phase of the introduction of stress factors. For example, it has been shown that ascorbate—the substrate for APX and a low molecular weight antioxidant itself—is rapidly depleted in chloroplasts when H_2_O_2_ is supplied [9]. The lack of ascorbate results in deactivation of ascorbate peroxidase and the failure of chloroplasts to neutralize H_2_O_2_. 

Furthermore, as described above, most antioxidant enzymes are located in the aqueous phases of the cell, while they are practically absent in the membranes. Considering that membranes contain vital electron transport chains—such as the photosynthetic electron-transport chain in the thylakoid membranes of chloroplasts and respiratory electron-transport chain in the inner mitochondrial membrane—the need to protect the components of these chains becomes obvious. In this regard, important low molecular weight antioxidants are isoprenoids and tocopherols. 

Low molecular weight antioxidants often perform not only antioxidant functions but also other functions in plant cells. For example, flavonoids are effective in controlling insect pests [10]. Ascorbate and tocopherols can improve nutritional properties of plants and seed quality. In addition, ascorbate is able to protect α-tocopherol from oxidation [11]. 

Thus, all the advantages described above underline the importance of altering non-enzymatic antioxidants to enhance the antioxidant capacity of plant cells, thereby reducing the oxidative status of plants under stress conditions. 

There are two primary approaches to increase the levels of low molecular weight antioxidants: (i) enhancing the expression of one or more genes encoding key enzymes involved in the biosynthesis of the target antioxidant; (ii) reducing the expression of genes encoding enzymes involved in the utilization or consumption of the specific antioxidant. These approaches can be achieved using both classical transgenesis and genome editing methods, either directly by altering the expression intensity of antioxidant biosynthesis genes or indirectly by regulating the activity of transcription factors for the corresponding genes.

The classical transgenesis technique involves the introduction of additional copies of the key enzyme-encoding genes into the plant genome, either from the same plant’s own genome or from other species. The method of classical transgenesis is widely used to create overexpressing lines, which are plants with significantly higher-than-normal expression of a specific gene. For this, additional copies of the gene are added, controlled by constitutive promoters which are always active, such as the cauliflower mosaic virus (CaMV) 35S promoter.

Classical transgenesis is also commonly used to create plants, notably *Arabidopsis thaliana* transgenic lines with knocked out genes, where T-DNA insertions, falling randomly into the plant’s own gene regions, disrupted their functions. Since insertion in these plants acts as a marker that allows cloning of DNA sections adjacent to the insertion, such plants are of interest for identifying genes, the function of which has been disrupted by the insertion. However, large-scale analyses of the composition of nucleotide sequences of extended DNA sections adjacent to the insertions revealed genetic disorders, such as translocations, inversions, and deletions, in some cases. These disorders can also act as sources of various phenotypic disorders and mask the phenotypic manifestations of mutations directly caused by T-DNA insertions. In addition, based on the results of a detailed study, in some cases, various disorders were detected away from the integration site of the foreign gene in such mutant plants. Additionally, this approach proved challenging in plant species lacking a wide range of knockout mutants, unlike Arabidopsis.

Another approach to regulate gene expression is RNA interference (RNAi). This method is based on the creation of genetic constructs with fragments of the coding region of the target gene linked to an antisense sequence. This promotes hairpin RNA formation after transcription, leading to the production of interfering RNAs that suppress gene expression at the post-transcriptional level. This is the so-called “gene knockdown” technique, which most often does not lead to complete suppression of gene expression. It is a convenient tool in cases where a partial reduction in gene expression is preferable, e.g., when a complete gene knockout could cause serious metabolic disorders.

The genome editing strategies, i.e., site-directed mutagenesis techniques, incorporate the use of zinc finger nucleases (ZFN), transcription activator-like effector nucleases (TALEN), and clustered regularly interspaced short palindromic repeats with CRISPR-associated protein (CRISPR/Cas) systems. CRISPR/Cas9 has become the most popular system due to its simplicity, accuracy, and versatility. This method uses ribonucleoprotein complexes that recognize targeted sequences in the genome. These complexes use synthetic guide RNA to direct a double-stranded DNA break. This break is then repaired either through homology-directed repair (HDR) using a donor template or through non-homologous end joining (NHEJ). The most common results of NHEJ are insertions and deletions (indels) in target sites, often leading to frame shift mutations in the coding sequence of the gene. This can result in the introduction of a stop codon, potentially leading to loss of gene function or, in some rare cases, to enhanced gene expression, for example, through a nonsense-mediated mRNA decay (NMD), by affecting alternative splicing (for review, see [12]). Thus, genome edited plants are increasingly displacing mutants with T-DNA insertions, and the CRISPR/Cas9 genome editing system has become widely used in applied research to create crops with valuable agronomic traits without introducing exogenous sequences and with minimal off-target changes in the genome [13,14]. 

Modern actively developing approaches designed on CRISPR/Cas9 provide the delivery of effector molecules or markers to certain DNA regions to change the transcriptional level of gene expression towards activation (CRISPRa) or suppression of gene activity (CRISPRi). A promising technique for creating mutants with altered gene expression is the use of Cas9 nuclease, which does not edit DNA but carries some additional functional elements. Cas9 proteins in which both nuclease domains are inactivated (deadCas9, dCas9) bind to DNA but cannot make cuts. Depending on where the dCas9:sgRNA binding site is located, transcription of the corresponding gene stops at either the initiation or elongation stage. By targeting gRNA to DNA regions where transcriptional repressors bind, the opposite effect can be achieved, i.e., activating gene expression. Another method of transcription regulation involves creating chimeric proteins by fusing dCas9 with eukaryotic transcription factors or the ω subunit of bacterial RNA polymerase. This provides an alternative approach for creating overexpressing lines with minimal introduction of foreign genes into the plant genome [12].

The present review describes the biosynthesis pathways of isoprenoid antioxidants, flavonoids, ascorbic acid, and glutathione. The mechanisms of the antioxidant activity of these compounds in relation to various ROS are also described. This review also emphasizes the success in developing a wide range of plants of different species with increased content of non-enzymatic antioxidants with deep analysis of the molecular genetic approaches used. Given the indispensable role of the CRISPR/Cas9 genome editing system in advancing functional genomics, we pay special attention to CRISPR/Cas9-based editing of plants. The most promising strategies for creation of crops with the efficient antioxidant properties and other valuable characteristics are summarized. The further development of these strategies may represent a universal approach for comprehensive qualitative improvement of plants under various environmental stresses.

## 2. Functioning of Non-Enzymatic Antioxidants in Higher Plant Cells and the Ways of Boosting Their Biosynthesis 

### 2.1. Isoprenoids

#### 2.1.1. Biosynthesis of Isoprenoids

**Figure 1:**
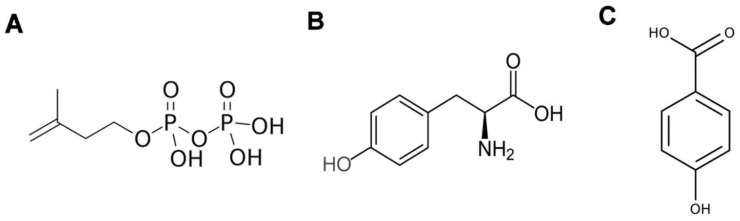
The chemical structures of the main precursors in isoprenoid synthesis. (**A**) Isopentenyl diphosphate; (**B**) L-tyrosine; (**C**) 4-hydroxybenzoic acid.

Isoprenoids (also known as terpenoids) belong to secondary metabolites, which include carotenoids, sterols, polyprenyl alcohols, ubiquinone-10 (UQ), plastoquinone-9 (PQ), tocopherols, and others. The first stages of their biosynthetic pathways involve the formation of prenyl side chain precursors and a benzoquinone ring. Isopentenyl diphosphate (IPP) (Figure 1A) is the universal isoprene precursor for prenyl side chain synthesis of all isoprenoids. A benzoquinone ring originates from L-tyrosine (Figure 1B) or phenylalanine, both products of the shikimate pathway, which is a specialized pathway for the biosynthesis of aromatic compounds. 

**Figure 2:**
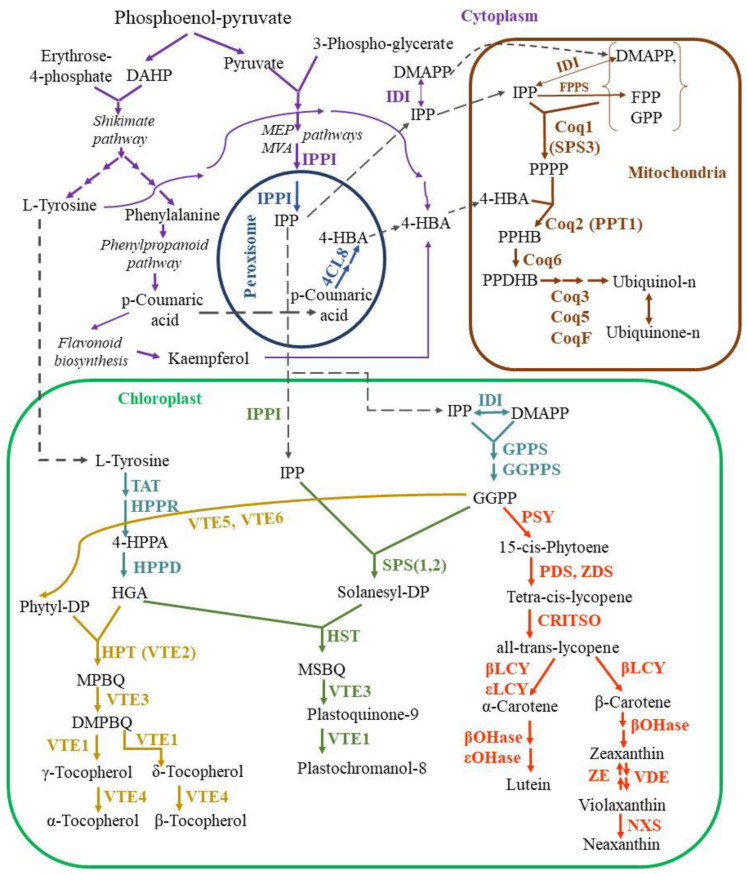
Schematic overview of biosynthetic pathways of isoprenoid antioxidants. Metabolites: 4-HBA, 4-hydroxybenzoic acid; 4-HPPA, 4-hydroxyphenylpyruvic acid; DAHP, 3-deoxy-D-arabino-heptulosonate 7-phosphate; DMAPP, dimethylallylpyrophosphate; DMPBQ, dimethyl-phytyl-benzoquinone; GGPP, geranylgeranyl diphosphate; HGA, homogentisate acid; IPP, isopentenyl diphosphate; MEP, 2C-methyl-D-erythritol-4-phosphate; MPBQ, methyl-phytyl-benzoquinone; MSBQ, methyl-solanesyl-benzoquinone; MVA, mevalonic acid; Phytyl-DP, phytyl diphosphate; PPDHB, polyprenyl-dihydroxybenzoate; PPHB, polyprenyl-hydroxybenzoate; PPPP, polyprenyl pyrophosphate; Solanesyl-DP, solanesyl diphosphate. Enzymes (colored): 4CL8, peroxisomal 4-coumarate CoA ligase; βLCY1, β-carotene cyclase; β-OHase, β-carotene hydroxylase; εLCY, ε- carotene cyclase; εOHase, ε-carotene hydroxylase; CoQ1 (SPS3), solanesyl diphosphate synthase; Coq3, Coq5, S-adenosyl-l-methionine (SAM)-dependent methyltransferases; Coq2 (PPT1), 4-hydroxybenzoate polyprenyl diphosphate transferase; Coq6, CoqF, flavin-dependent monooxygenases; CRTISO, carotenoid isomerase; FPPS, farnesyl diphosphate synthase; GGPPS, geranylgeranyl diphosphate synthase; GGPPS, geranylgeranyl diphosphate synthase; HPPD, 4-hydroxyphenylpyruvate dioxygenase; HPPR, 4-hydroxyphenylpyruvatereductase; HPT (VTE2), homogentisate phytyl transferase; HST, homogentisate solanesyl diphosphate transferase; IDI, isopentenyl diphosphate isomerase; IPPI, isopentenyl diphosphate isomerase; NXS, neoxanthin synthase; PDS, Phytoene desaturase; PSY, Phytoene synthase; SPS, solanesyl diphosphate synthases; TAT, tyrosine aminotransferase; VDE, violaxanthin de-epoxidase; VTE1, tocopherol cyclase; VTE3, MPBQ/MSBQ methyl transferase; VTE4, γ-tocopherol methyltransferase; VTE5, phytol kinase; VTE6, phytyl-phosphate kinase; ZDS, ζ-carotene desaturase; ZE, zeaxanthin epoxidase. The enzymes of the cytoplasmic stages of isoprenoid synthesis are marked in purple, the stages and enzymes of the chloroplast isoprenoid synthesis are marked in turquoise, the stages of tocopherol synthesis are yellow-brown, the stages of plastoquinone synthesis are green, and the stages of carotenoid synthesis are red. The stages and enzymes of ubiquinone synthesis are marked in brown.

In the second stage, the condensation of the ring and the prenyl side chain with subsequent modifications takes place [15]. The prenyl side chain is synthesized in the cytoplasm IPP, which is converted by isopentenyl diphosphate isomerase (IPPI) to dimethylallyl pyrophosphate (DMAPP), followed by the synthesis of numerous isoprenoids, including PQ and UQ (Figure 2). Information about the genes which encode the main enzymes of the isoprenoid biosynthesis in *Arabidopsis thaliana* is given in Table 1.

The transformation of IPP to DMAPP occurs both in the stroma of chloroplasts and in the matrix of mitochondria with the involvement of polyprenyl diphosphate synthase (PPS), such as geranyl diphosphate synthase (GPPS), farnesyl diphosphate synthase (FPPS), and geranylgeranyl diphosphate synthase (GGPPS) [38,39]. These diphosphate synthases catalyze the formation of polyprenyl diphosphates with various chain lengths, making them the key enzymes in the biosynthesis of many isoprenoid compounds, such as PQ and UQ, vitamin E, carotenoids, and others [40].

##### Ubiquinone Synthesis

For the synthesis of UQ, IPP is transported to mitochondria, where further stages of UQ synthesis are carried out. In plants, the isoprene subunits for the UQ side chain are generated through the mevalonate (MVA) pathway, which takes place both in cytoplasm and peroxisomes (Figure 2). The produced precursors are further used for the biosynthesis of sesquiterpenes, triterpenes, sterols, and brassinosteroids [41,42]. The next step of UQ synthesis is the formation of polyisoprenoid tail of polyprenyl pyrophosphate (PPPP) in mitochondria by solanesyl diphosphate synthase (SPS3), which is trans-polyprenyl diphosphate synthase, also called Coq1. This is one of the most important enzymes in UQ synthesis, since Arabidopsis AtSPS3 knockout mutants were embryo-lethal [20]. The most likely substrate for SPS3 in plants is farnesyl diphosphate (FPP), as evidenced by the reduced UQ level in loss-of-function FPP synthase mutants FPS1 and FPS2 [43].

The aromatic ring precursor for UQ biosynthesis is 4-hydroxybezoic acid (4-HB, Figure 1C), which is derived from either L-phenylalanine or L-tyrosine. Phenylalanine is converted to p-coumaric acid through the phenylpropanoid pathway, which also precedes the synthesis of flavonoids in cytoplasm. Formed in this way, flavonoid kaempferol serves as one of the 4-HB sources [44]. The other pathway involves the conversion of p-coumaric acid into 4-HB by β-oxidative metabolism, in which p-coumaric acid is imported into peroxisomes [45]. In peroxisomes, 4-coumarate CoA ligases (AT4G19010 and 4CL8) catalyze the formation of p-coumaroyl-CoA with subsequent 4-HB formation. 

Coq2, 4-hydroxybenzoate polyprenyl diphosphate transferase (PPT1), is another important enzyme in UQ biosynthesis, which provides the catalysis of its rate-limiting stage [15]. PPT1 transfers the polyisoprenoid chain of PPPP to the 4-HB ring, generating the first lipophilic UQ intermediate, polyprenyl-hydroxybenzoate (PPHB) [21]. Further UQ biosynthesis requires subsequent hydroxylation of C1, C5, and C6 positions of the aromatic ring in PPHB structure catalyzed by flavin-dependent monooxygenases (Coq6 and CoqF) and S-adenosyl-l-methionine-dependent methyltransferases (Coq3 and Coq5) [46]. The co-expression of the genes involved in UQ biosynthesis in mitochondria as well as of the genes involved in 4-HB biosynthesis and the MVA pathway was shown in Arabidopsis [20,47]. In the context of plants, both the regulation of UQ biosynthesis and the response of the genes encoding UQ biosynthetic enzymes to environmental stimuli are less studied than in yeasts and mammals [46]. 

##### Plastoquinone Synthesis

The synthesis of the benzoquinone ring for all isoprenoids synthesized in chloroplasts, as described above, originates from L-Tyrosine (Figure 2). The resulting L-Tyrosine is converted by 4-hydroxyphenylpyruvate dioxygenase (HPPD) to homogentisate acid (HGA) via 4-hydroxyphenylpyruvic acid (HPPA). It is HGA that is the benzene quinone ring precursor for PQ and tocopherols in plants. This is an important step in the synthesis of plastid tocopherols in plants. The second key component in PQ synthesis, solanesyl diphosphate, is synthesized from geranylgeranyl diphosphate (GGDP) and IPP by a reaction catalyzed by solanesyl diphosphate synthase (SPS). This is one of the most important regulatory enzymes in PQ synthesis, since under exposure of Arabidopsis plants to increased light intensity, an accumulation of PQ was observed accompanied by the increase of transcript levels of the genes encoding the enzymes of plastoquinone biosynthesis and, first of all, the gene encoding SPS1 [48]. Condensation of HGA ring and side chain of solanesyl diphosphate is the first direct step in PQ synthesis via a methyl-solanesyl-benzoquinone intermediate. The formation of PQ and its product plastochromanol-8 (hereafter referred to as plastochromanol) occurs by tocopherol cyclase enzymes (VTE3 and VTE1). 

##### Tocopherol Synthesis

VTE3 and VTE1 are also involved in the biosynthesis of γ- and δ-tocopherols (Figure 2). Phytyl-PP for tocopherols synthesis originates from GGDP by phosphorylation of its free phytol with VTE5 or VTE6. Both of these enzymes are important for vitamin E synthesis: tocopherol levels reduced to 20% in *VTE5* knockout Arabidopsis plants, compared to the wild-type plants [32]. Phytol phosphorylation, catalyzed by VTE6 was also shown to be important for phylloquinone biosynthesis, which in turn is required for Photosystem I (PS I) complex functioning and stability [33]. The VTE6 knockout Arabidopsis mutants exhibited an impaired function of PS I due to a higher rate of PS I subunits degradation and increased PS I susceptibility to photodamage, compared to the wild-type plants.

Phytyl-PP molecules are condensed with HGA by homogentisate prenyl transferase (HPT, VTE2) to yield 2-methyl-6-phytylplastoquinol (MPBQ), which is methylated by VTE3 to form 2,3-dimethyl-5-phytyl-1, 4-benzoquinone (DMPBQ). MPBQ and DMPBQ are substrates for VTE1 to yield δ- and γ-tocopherols (Figure 2). Finally, γ-tocopherol methyltransferase (VTE4) converts δ- and γ-tocopherols (and tocotrienols) to β- and α-tocopherols. HPT is the enzyme which catalyzes the limiting step in tocopherols biosynthesis [49]. 

##### Carotenoid Synthesis

Carotenoid synthesis is also a branch of terpenoid plastid biosynthesis from the same precursors as for tocopherols and PQ (Figure 2). Carotenoid synthesis starts from the condensation by the enzyme phytoene synthase (PSY) of two GGDP molecules to produce phytoene. Two desaturases, phytoene desaturase (PDS) and ζ-carotene desaturase (ZDS), catalyze similar dehydrogenation reactions by introducing four double bonds to form tetra-cis-lycopene. 

The knockout of the *PDS* gene in many crop species resulted in a phenotype with pigmentation loss. Albino regenerants of cells with edited *PDS* gene have been obtained for *Malus domestica* plants, for diploid and octoploid strawberries, yams, and onion [50,51,52,53]. The regenerants of carrot cells with mutations in the region of the *DcPDS* and *DcMYB113*-like genes were depigmented [54].

Desaturation requires a plastid terminal oxidase and plastoquinone in photosynthetic tissues [55,56,57]. In the next step, the carotenoid isomerase (CRTISO) catalyzes cis–trans isomerization resulting in all-trans-lycopene. This enzyme is important for optimal carotenoid synthesis in etioplasts, chromoplasts, and chloroplasts [58]. Mutant plants deficient in CRTISO activity accumulate various cis-isomer biosynthetic intermediates when grown in the dark, but these intermediates can be photoisomerized in the light and yield viable plants, albeit with reduced lutein levels [24]. The next steps of carotenoid biosynthesis split into two main branches differing by cyclic end-groups. One branch is responsible for the synthesis of α-carotene and its derivatives by lycopene epsilon cyclase (εLCY) and lycopene beta cyclase (βLCY). Their altered expression in mutants resulted in lutein levels ranging from 10% to 180% of those in the wild-type plants [59]. With participation of βLCY, lycopene is cyclized to introduce β-ionone, which leads to the other β,β branch of carotene synthesis. The functioning of this branch leads to the synthesis of β-carotene and its derivatives, provitamin A and the components of the xanthophyll cycle, violaxanthin, antheraxanthin, and zeaxanthin. 

The *Orange genes* (*Or*), which were found in many plant species, are a class of regulatory genes that mediate carotenoid accumulation [60]. It has been shown that, in sweet potato, the product of *IbOr* can protect PSY and the oxygen-evolving enhancer protein PsbP of photosystem II (PS II) [61,62]. The product of this gene does not directly participate in carotenoid biosynthesis but is involved in chromoplast differentiation, creating a storage site for carotenoids [63]. Two spontaneous natural mutations in the *Or* were recognized in *Brassica oleracea*, which were responsible for the orange color of the inflorescences [64]. The overexpression of the *Or* leads to an increase in carotenoid content in transgenic potatoes [65] and maize [66].

During fruit ripening, a signal transduction cascade in response to the plant hormone ethylene involves proteins of the ETHYLENE-INSENSITIVE 3/ETHYLENE-INSENSITIVE 3-LIKEs (EIN3/EILs) family. In tomato plants, CRISPR/Cas9 knockouts of *eil2* were shown to produce yellow or orange fruits, in contrast to the red wild-type tomato fruits. Further analysis of the transcriptome and metabolome data of the ripe fruits of the mutant and the wild-type showed that TF EIL2 is involved in the accumulation of β-carotene through direct regulation of the expression of the SlERF.H30 and SlERF.G6 genes, which, in turn, are involved in the regulation of the βLCY gene in tomatoes (SlLCYB2) [67].

Thylakoid lumen acidification under high light conditions activates violaxanthin de-epoxidase, resulting in zeaxanthin formation from violaxanthin through antheraxanthin [68]. The interconversion reaction, which is the epoxidation of zeaxanthin by zeaxanthin epoxidase localized on the stromal side of the thylakoid membrane, takes place under low light intensities [29]. Conversion of violaxanthin to neoxanthin is catalyzed by neoxanthin synthase (NXS). 

Fibrillins are lipid-associated proteins which are known as structural components of carotenoid sequestering in plant chromoplasts [69,70]. Some fibrillins have the ability to bind proteins involved in the synthesis of plastid isoprenoids, thus contributing to their activity. Fibrillin 5 (FBN5) was found to be essential for PQ biosynthesis. FBN5 stimulates enzymatic activity of SPS1 and SPS2 through binding to its solanesyl moiety [71]. The same authors have shown that homozygous Arabidopsis mutants with the FBN5 encoding gene knocked out were seedling-lethal, and the growth rate of transgenic plants with low FBN5-B levels was slower than that of wild-type plants. FBN6 was shown to interact with PSY and increase its enzymatic activity [72].

#### 2.1.2. Activity of Isoprenoids towards ROS

Quinones, both plastoquinone, the mobile electron carrier in the photosynthetic electron-transport chain of the chloroplast, and ubiquinone, the mobile electron carrier in the respiratory electron-transport chain of the mitochondria, possess efficient antioxidant activity. The fully reduced PQ, plastohydroquinone (PQH_2_), and UQ, ubihydroquinone (UQH_2_), neutralize superoxide anion radical (O_2_^•−^), protecting against lipid peroxidation of the membranes during oxidative stress conditions of both abiotic and biotic nature [73,74,75,76,77]. It is known that O_2_^•−^, especially in the protonated state (perhydroxyl radical, HOO^•^) initiates lipid peroxidation [76,78]. 

O_2_^•−^ is the primary singly reduced product of molecular oxygen reduction. In thylakoids, O_2_^•−^ is generated at the level of PS I outside the membrane by F_A_/F_B_ terminal clusters and to a high extent inside the thylakoid membranes by phyllosemiquinone (the singly reduced phylloquinone or vitamin K) [4,79] presumably in A_1_ sites under stress conditions [80]. Moreover, O_2_^•−^ can be produced in the plastoquinone pool (PQ pool) [81] as well as in some other complexes of the chain under specific conditions (reviewed in [80]. In the PQ pool, O_2_^•−^ is produced in the reaction of singly reduced PQ, plastosemiquinone (PQ^•−^), with molecular oxygen. Presumably, PQ^•−^ appears in the PQ pool owing to comproportionation of PQ and PQH_2_ molecules rather than as the result of PQH_2_ oxidation in the plastohydroquinone-oxidizing site of cytochrome *b6/f* complex (reviewed in [82]).

In the mitochondria, the electron transfer to molecular oxygen with generation of O_2_^•−^ can proceed in complex I and complex III. In complex I, such a reducing agent is presumably reduced flavin mononucleotide [83], and at the level of complex III, it is believed to be a singly reduced ubiquinone, ubisemiquinone (UQ^•−^), formed as a result of the oxidation of UQH_2_ in the quinol-oxidizing site of complex III [84]. Meanwhile, it is possible to predict that in the UQ pool, as in the PQ pool, UQ^•−^ may also be produced owing to comproportionation of UQ and UQH_2_.

Reactions of the reduced quinones with O_2_^•−^ were studied in detail; in an aqueous medium, the constant rate of second order reaction is estimated to be around 10^8^ M^−1^ s^−1^ [85], and around 10^4^ M^−1^ s^−1^ in acetonitrile [86]. We propose that the reaction of PQH_2_ with O_2_^•−^ as well as the reaction of UQH_2_ with O_2_^•−^ occurs predominantly at the membrane/water phase boundary, where the rate constant of 10^8^ M^−1^ s^−1^ is applicable. We have previously estimated that the equilibrium constant of the reaction of PQH_2_ with O_2_^•−^ should be equal to or even higher than 10^9^ [82]. 

The reaction of the reduced quinone with O_2_^•−^ results in the formation of H_2_O_2_ [77,87], which possesses a lower reactivity among ROS. It has now been proven that PQH_2_ is also able to react with H_2_O_2_ [86,88,89]; however, the rate constant of the reaction of hydroquinones with H_2_O_2_ is rather low, approximately 10^3^ M^−1^ s^−1^–10^4^ M^−1^ s^−1^ in the phosphate buffer [88].

Another aspect of the antioxidant activity of the reduced quinones is related to their ability to quench singlet oxygen, ^1^O_2_, which is known to initiate lipid peroxidation as well. ^1^O_2_ is formed as a result of the spin inversion of one of the unpaired electrons in the O_2_ molecule. This is especially relevant to chloroplasts, where the main way of ^1^O_2_ generation is the energy transfer from the chlorophyll in triplet state (^3^Chl) to O_2_. That process primarily occurs in PS II of the photosynthetic electron-transport chain in thylakoids [90,91]. The energy of ^3^Chl is approximately 1.3 eV, allowing the molecular oxygen to be converted into ^1^O_2_ (~1 eV).

PQH_2_ neutralizes ^1^O_2_ [86,92,93] owing to either chemical or physical mechanisms with a rate constant of approximately 10^8^ M^−1^ s^−1^ [94,95]. The physical mechanism of quenching is based on the energy transfer from ^1^O_2_ to PQH_2_, resulting in conversion of ^1^O_2_ back to O_2_, while the chemical mechanism of quenching is due to oxidation of PQH_2_ (presumably of its isoprenoid chain), resulting in generation of the plastoquinone derivatives, hydroxyplastoquinones. 

The ability to quench ^1^O_2_ is also typical for other prenylquinols, including tocochromanols such as tocopherols, tocotrienols, and plastochromanol [95]. Tocopherols in organic solvents can neutralize both O_2_^•−^ with the rate constant of 10^6^ M^−1^ s^−1^ [96] and ^1^O_2_ with the rate constant of 10^8^ M^−1^ s^−1^ [94], as well as scavenge OH^•^ [97] and decompose H_2_O_2_ [98]. 

Although the reaction of UQH_2_ with ^1^O_2_ is less studied, it can be assumed that this activity is also characteristic of ubihydroquinone and both mechanisms, physical and chemical, can be applied.

Quinones may not only directly quench ^1^O_2_ but also quench ^3^Chl, therefore preventing the formation of ^1^O_2_ [99]. Carotenoids, the tetraterpenoid organic pigments, exert an antioxidant activity through the same pathway, i.e., due to their ability to quench both ^1^O_2_ and ^3^Chl. Carotenoids are subdivided into two main groups. The first group is xanthophylls, which are the oxygenated carotenoids. The representatives of this group in thylakoids are violaxanthin, zeaxanthin, lutein, neoxanthin, fucoxanthin, etc. The second group is carotenes; in thylakoids these are β-carotene and lycopene. Quenching of ^3^Chl by carotenoids mainly proceeds in the antenna system of PS II [100,101]. Alboresi et al. [102] showed that the lack of lutein and zeaxanthin resulted in a higher generation of ^1^O_2_ in thylakoids. Telfer [100] suggested that the main function of β-carotene of the PS II reaction center is the quenching of ^1^O_2_ if it is generated there.

Carotenoids, similar to quinones, quench singlet oxygen by physical and chemical mechanisms, and it has been shown that chemical quenching is much weaker than physical quenching [103]. The rate constants of physical quenching of ^1^O_2_ by β-carotene and zeaxanthin were estimated to be 7 × 10^9^ M^−1^ s^−1^ and of 7 × 10^8^ M^−1^ s^−1^ for fucoxanthin, while the rate constants of chemical quenching for β-carotene and zeaxanthin were approximately 4 × 10^6^ M^−1^ s^−1^ and 3 × 10^5^ M^−1^ s^−1^ for fucoxanthin [103]. Similar results were presented in [104]. 

However, there is a series of works in which, using biochemical and biophysical methods, researchers have shown that some carotenoids can be involved in the formation of singlet oxygen themselves; for example, Ashikhmin et al. [105] provided the evidence that phytofluene, the uncolored C40 carotenoid with a short chain, effectively generated ^1^O_2_ under UVA conditions, while it was able to quench ^1^O_2_ in the dark. 

#### 2.1.3. Genetic Approaches for Boosting Isoprenoid Production in Plants 

The biosynthetic pathway of isoprenoid synthesis involves numerous enzymes (see Table 1 and Figure 2). For many of these enzymes, the impact of overexpression or knockout on the content of various isoprenoids and the characteristics of the mutant plants have been studied. The regulation of isoprenoid biosynthesis is a complex, multi-level process, including transcriptional, epigenetic, and post-translational control. At each of these stages, it is possible to interfere with genetic engineering methods. The content of isoprenoids in plants can be changed using different approaches, such as introducing additional gene cassettes, which affect the intensity of enzyme biosynthesis or by introducing targeted mutations into the genes of these enzymes.

One of the approaches for the elevation of the level of various isoprenoids is upregulation of the expression of the genes encoding the enzymes of the initial steps of the isoprenoid biosynthetic pathway. However, such an approach may be associated with certain challenges, since the correspondent metabolites are used in various branches of the biosynthesis of antioxidants further downstream. For instance, Arabidopsis plants overexpressing the FPPS encoding gene (see Figure 2) exhibited lower levels of endogenous isoprenoids compared to the wild-type plants [106]. Moreover, these plants displayed necrotic damage accompanied by an increase in hydrogen peroxide accumulation, especially under constant illumination. It appears that elevation of *FPPS* expression leads to metabolic disruptions, possibly due to the rapid depletion of IPP (isopentenyl diphosphate), a precursor for all isoprenoids.

Another enzyme, HPPD, plays a crucial role in the metabolic pathway leading to the synthesis of tocopherols, PQ, and plastochromanol. Studies involving overexpression of the *HPPD* gene yielded contradictory results. For example, overexpression of *HPPD* in Arabidopsis led to an approximately 40% increase in tocopherol accumulation in leaves [107]. In tobacco plants with overexpression of the barley *HPPD* gene under the 35S promoter, there was no increase in tocopherol content in the leaves; however, tocopherol levels increased in seeds [108]. Interestingly, these tobacco plants exhibited a significant (up to 50% of wild-type levels) increase in PQ content in leaves [107]. Transgenic sweet potato plants overexpressing HPPD were found to be more resistant to drought (cessation of watering for 14 days), salinity (watering with 200 mM NaCl), and oxidative stress (induced by incubating leaf discs in 5 µM methyl viologen) compared to non-transgenic plants [109]. Similar to the findings in Falk et al. [108], the authors of that study did not observe an increase in tocopherol content in the leaves of transgenic sweet potatoes. Unfortunately, the authors did not analyze the content of plastoquinones and plastochromanol, so the mechanism of resistance of these transgenic plants remains unclear. 

Nevertheless, when enzymes are exclusively involved in the biosynthesis of specific types of isoprenoids, successful genetic transformations have been reported. For example, in Arabidopsis, the overexpression of the gene encoding peroxisomal 4CL involved in the oxidation of p-coumaric acid for the subsequent biosynthesis of UQ (Figure 2) led to an approximately 1.5- to 2-fold increase in UQ accumulation [16,17].

The most promising approaches to enhance isoprenoid content involve genetic engineering manipulations with enzymes catalyzing the last steps of biosynthesis of certain antioxidants of interest. Overexpression of *VTE1*, which catalyzes both the penultimate step in tocopherol biosynthesis and the conversion of PQ to plastochromanol (Figure 2), resulted in a seven-fold increase in tocopherol accumulation in Arabidopsis leaves [110]. Another study involving *VTE1*-overexpressing Arabidopsis plants demonstrated a significant accumulation of plastochromanol in leaves, approximately 60 times higher than in wild-type plants [111]. Tobacco plants overexpressing the *VTE1* gene from Arabidopsis exhibited increased resistance to drought stress, with reduced lipid peroxidation and H_2_O_2_ content under stress conditions [112]. Rice plants overexpressing *VTE1* (using a construct with two 35S promoters) demonstrated enhanced resistance to salinity stress, along with decreased H_2_O_2_ levels under stress conditions [113].

It is known that α-tocopherol exhibits the highest biological activity. That is why many studies focus on the strategy of increasing the content of this form of tocopherol in plants. The enzyme VTE4 is involved in the final stage of α-tocopherol biosynthesis (Table 1, Figure 2). It has been used extensively for genetic engineering modifications of various plant species, including the increase of α-tocopherol content in soybean seeds to enhance their nutritional value. The expression of the gene encoding VTE4 from *Perilla frutescens* under a seed-specific promoter (vicilin) resulted in a ten-fold increase in α-tocopherol content in soybean seeds [114]. Overexpression of *AtVTE4* in soybean seeds showed a four-fold increase in α-tocopherol content [115], although the overall tocopherol content changed only slightly.

In the study by Li et al. [116], screening was conducted to assess the impact of overexpressing various tocopherol biosynthesis genes, both separately and in certain combinations (*VTE2* + *VTE4* and *VTE3* + *VTE4*), on tocopherol content. The highest accumulation of tocopherols in the form of α-tocopherol occurred in Arabidopsis plants with simultaneous overexpression of *VTE2* and *VTE4*, approximately six times higher than in the wild-type plants. Additionally, a high level of tocopherol accumulation was demonstrated in transgenic Arabidopsis plants with increased VTE2 content [116]. Interestingly, transgenic *Codonopsis lanceolata* plants overexpressing the Arabidopsis gene *AtVTE4* exhibited a higher antimicrobial activity against *Staphylococcus aureus* and *E. coli* compared to the wild-type plants [117]. The increased antimicrobial activity may be associated with the elevated accumulation of tocopherols in these transgenic plants that was approximately six times higher than in the leaves of wild-type plants. 

Because UQ is a component of the respiratory chain common to all eukaryotes, gene transfer from other eukaryotic organisms, such as yeast, into plant genomes is feasible. The expression of *CoQ2* from yeast led to a six-fold increase in ubiquinone content in tobacco leaves [118]. The resulting transgenic plants showed increased resistance, compared to the wild-type plants, against salinity stress (300 mM NaCl) and oxidative stress induced by the addition of 50 µM methyl viologen [118]. 

Overexpression of the SPS enzymes (Figure 2) in different plant species has led to increased accumulation of UQ or PQ, depending on the specific variant of the *SPS* gene used. In *Salvia miltiorrhiza* plants, the overexpression of the *SmPP1* gene resulted in approximately a 2-fold increase in PQ content, while the overexpression of the *SmPP2* gene led to a 1.5-fold increase in UQ content [119].

In Arabidopsis plants with the overexpression of *SPS1*, the content of PQ increased approximately 1.5- to 2-fold, and the content of plastochromanol increased approximately 3-fold compared to that in wild-type plants [48]. These transgenic plants showed greater resistance to high light conditions (1300 µmol photons/m^2^ s) compared to the wild-type plants: they exhibited decreased lipid peroxidation and PS II photoinhibition. Since there was a significant increase in plastochromanol content in these plants, it can be speculated that the increased resistance is determined by the higher plastochromanol content. However, despite the similarly increased plastochromanol content, the plants overexpressing *VTE1* did not differ from wild-type plants in their resistance to high light conditions [48]. Furthermore, plants with a *VTE1* knockout, which were highly sensitive to increased light conditions, regained their resistance to light stress when *SPS1* was overexpressed in these plants [120]. Therefore, increasing PQ content in leaves by genetic engineering approaches could be a promising way to enhance plant sustainability under photoinhibitory conditions. 

As detailed above, carotenoids also play an important antioxidant role. Furthermore, β-carotene serves as a precursor to vitamin A, which humans cannot synthesize on his own and must obtain it from food. Both of these factors explain the numerous attempts to increase the content of carotenoids in the leaves and fruits of plants. 

One of the best-known examples of plants with increased β-carotene content is “Golden Rice”, created through the simultaneous overexpression of the narcissus gene encoding PSY (Figure 2) and a bacterial carotene desaturase [121]. Subsequently, “Golden Rice II” was developed, using the *PSY* gene from maize, resulting in a 23-fold increase in carotenoid content compared to the original “Golden Rice” (Table 1) [122]. Seeds of *Brassica napus*, known as “Golden Canola”, were generated through the overexpression of a bacterial *phytoene synthase* gene, leading to carotenoid levels in these seeds approximately 50 times higher than in non-transgenic seeds [123]. Similar approaches were used to produce “Golden Maize” seeds [124]. In transgenic bananas, the highest accumulation of carotenoids was detected in lines carrying the banana *PSY* gene rather than the maize *PSY*. In these bananas, the carotenoid content reached 55 µg/g, exceeding that in “Golden Rice II” seeds [125].

An alternative approach for obtaining plants with increased β-carotene content involves inhibiting the activity of enzymes that use β-carotene as a substrate. Orange plants with a significant (up to 36 times) increase in β-carotene content were generated through the suppression of β-carotene hydroxylase (*β-OHase*) expression using RNA interference (Figure 2, Table 1) [126]. The antioxidant properties of these oranges were tested on a model organism, a small nematode worm *Caenorhabditis elegans*. The study revealed that the survival rate of worms under oxidative stress conditions (hydrogen peroxide supplementation) increased by 20% when they were fed oranges with enhanced β-carotene content [126]. 

To increase the β-carotene content in sweet potatoes, RNA interference of *β-OHase* was also employed to suppress its expression level [127]. The total carotenoid content in these transgenic plants increased by 10 to 18 times, β-carotene content increased by 16 to 35 times, and the amount of zeaxanthin increased by 5 to 15 times. The resulting plants demonstrated increased resistance to salinity (150 mM NaCl) and reduced accumulation of ROS under stress conditions [127]. 

On the other hand, overexpression of *β-OHase* in Arabidopsis only led to a slight reduction in the amount of β-carotene due to its conversion into xanthophylls, along with a significant accumulation of xanthophyll carotenoids, reaching up to 40% of the total carotenoid content under moderate light conditions [128]. These transgenic Arabidopsis plants showed increased resistance to higher light intensity and elevated temperatures (1000 μmol photons/m^2^ s, 40 °C) [128], which is in line with the role of xanthophylls in protecting plants against photoinhibition. Additionally, the transgenic Arabidopsis plants accumulated significantly lower amount of anthocyanin (see below), which also serves as a stress indicator for Arabidopsis. The levels of lipid peroxidation in the leaves of transgenic plants were also reduced under high light conditions compared to wild-type plants [128].

Mulberry plants overexpressing *β-OHase* under the 35S promoter displayed increased resistance to UV radiation, high light (1000 μmol photons/m^2^ s), and heat stress (40 °C), compared to wild-type plants, resulting in reduced accumulation of ROS in the leaves [129]. In Lisianthus plants, overexpression of the Arabidopsis gene encoding β-OHase also led to a significant increase in carotenoid content (1.5–3 times) and, notably, enhanced accumulation of zeaxanthin (1.5–2 times) [130]. These transgenic plants were also more resistant to light stress [130]. Another way to increase zeaxanthin content in plants is by gene silencing of *zeaxanthin epoxidase* (see the carotenoid biosynthesis pathway in Figure 2). For example, using this approach on potato plants resulted in the growth of zeaxanthin content in tubers up to 130 times greater compared to the control plants [131].

LCYs are the key enzymes in the carotenoid biosynthesis pathway in higher plants (Table 1, Figure 2). Therefore, in many experimental studies on various plant species, LCY encoding genes are the targets for genome editing in order to increase the accumulation of bioactive lycopene. Tomato plants with a five-fold increase in lycopene content in fruits compared to unedited plants were transformed using the multiplex editing system CRISPR/Cas9. This approach resulted in simultaneous knockout of five genes: *Stay-green 1* (*SGR1*) gene for the stimulation of lycopene synthesis, *εLCY*, and three *βLCY* genes, preventing cyclization of lycopene. Single, double, triple, and quadruple mutants of these genes were characterized, with the highest accumulation of lycopene in fruits for *SGR1* single mutants [132]. Reducing the activity of εLCY should lead to an increase in β-carotene and zeaxanthin, but it may also result in a decrease in lutein levels that could have negative consequences for plants. There are mentions in the literature about the specific reduction of εLCY activity in tubers from potato plants obtained using antisense sequences of the *εLCY* gene fragments introduced under the control of patatin promoter, which is specific for tuber tissues. In the resulting transgenic plants, carotenoid content in the tubers increased 2.5 times, and β-carotene content increased 14-fold [133]. In order to increase the provitamin A content, the fifth exon of the *εLCY* gene was transformed in an embryogenic cell suspension of commercial banana varieties, specifically the Cavendish, Grand Naine, and Rasthali cultivars, followed by regeneration of transformed plants. The β-carotene content in the flesh of mature fruits of edited banana lines was six times higher compared to unedited fruits, while the amounts of lutein and α-carotene were reduced [134].

Using CRISPR/Cas9 genomic technology, a single His in position 523 was replaced by Leu in εLCY in rice callus culture [135]. For precise targeted mutagenesis, a matrix delivery system based on a geminiviral replicon was used, which made it possible to significantly increase the frequency of homologous recombination and to obtain the transformed rice calli with a bright orange color with a success rate of 1.32%. The total content of carotenoids in the resulting edited callus lines was 6.8–9.6 times higher than in the wild-type callus. The authors also showed a decrease in the accumulation of ROS in the edited lines under salt stress conditions. Unlike traditional methods that rely on randomly selecting mutations and monitoring their effects, this work specifically targeted the *εLCY* gene for precise editing. It was performed as a follow-up to the study by Ishihara et al. [136], who found that a polymorphism of one nucleotide in the *εLCY* gene (*H523L*) led to an increase in the accumulation of carotenoids in rice calli. This experiment demonstrates the possibility of the replacement of the genes by “elite alleles” within one generation, which makes an invaluable contribution to the development of new varieties of agricultural plants.

*Nicotiana tabacum* K326 plants, which are the knockouts of the homologous genes encoding εLCY, *Ntε-LCY1* and *Ntε-LCY2*, were obtained using CRISPR/Cas9 by Song et al. [137]. The authors studied the phenotypes of the mutants, the expression pattern of carotenoid biosynthesis pathway genes, and the response to light stress. This made it possible to identify functional differences in the expression of homologues. Mutations in the *Ntε-LCY* gene regions led to an increase in growth rate of leaves, an accumulation of carotenoids and chlorophyll, and an increase in stress resistance to strong light of tobacco plants. All these effects were most pronounced in *Ntε-LCY2* mutant plants [137].

The use of the Target activation induced cytidine deaminase (Target-AID) technology results in obtaining the alleles of the *DNA damage UV binding protein 1* (*SlDDB1*)*/de-etiolated1* (*SlDET1*) genes, and the genes encoding βLCY (*SlCYC-B*) that affects the accumulation of carotenoids in tomatoes. The content of carotenoids, lycopene and β-carotene in the edited lines was higher compared to the wild-type tomato plants [138,139]. This study showed the possibility of simultaneous replacement of nucleotides in several target genes in one plant within one generation, that has potential advantages in breeding work.

Another promising approach to increase the content of carotenoids in plants is the expansion of storage space for carotenoids together with intensification of their biosynthesis. The previously described *Or* gene is not only a chaperone capable of binding to PSY, but is also involved in chromoplast formation, a storage site for carotenoids [63]. This gene encodes the cysteine-rich protein DnaJ and regulates the accumulation of carotenoids in chromoplasts [140,141]. The use of the CRISPR/Cas9 system makes it possible to introduce targeted mutations into various parts of the *Or* gene and to identify the parts of the gene where the changes would lead to an increase in carotenoid accumulation. A model cell line of orange rice callus was obtained by targeted mutagenesis of the *OsOr* gene. The authors showed that it was the single guide RNA targeting region between the third exon and the third intron which produced the orange calli phenotype. The transformed cell line accumulated more lutein and β-carotene compared to the wild-type callus line and was characterized by an increase in the level of transcripts of the genes encoding the enzymes of the carotenoid metabolic pathway: PSY2, PSY3, PD, ZDS, and βLCY. Orange callus plants also showed increased tolerance to salt stress [142]. 

An interesting and promising approach involves simultaneously overexpressing genes in the isoprenoid biosynthesis pathway that are located upstream of the target product and reducing the activity of downstream enzymes. For example, the knockdown of the activity of β-OHase in wheat endosperm led to an approximately 10-fold increase in β-carotene content [143]. Overexpression of the *PSY* gene resulted in an approximately 14-fold increase in β-carotene content. As a result, the simultaneous use of both of these strategies led to an approximately 30-fold increase in β-carotene content in wheat endosperm [143].

There are examples of successful editing of genes encoding the proteins of isoprenoid catabolism resulting in an increase in isoprenoid content. One such example is the editing of *Carotenoid cleavage dioxygenases* (*CCDs*), which belong to a small family of genes that play an important role in carotenoid degradation. Silencing of the *CCD4* gene using CRISPR/Cas9 in banana plants resulted in an increase of β-carotene content of 1.3–1.4 times in leaves and of 2.3–2.7 times in roots compared to the wild-type plants [144]. 

A new step in plant biotechnology involved the production of marker-free rice plants with an increased content of carotenoids in grains by the insertion of a biosynthesis gene cassette into two specially selected regions of the plant genome, called genomic safe harbors (GSHs). The integration of genetic constructs into GSHs avoids negative consequences of accidental random insertions into vital regions of the plant genome. The carotenoid biosynthesis gene expression cassette included the carotenoid desaturase gene from *Erwinia uredovora* (SSU-crtI) and *maize phytoene synthase* (*ZmPsy*) under the control of the endosperm-specific glutelin promoter [145]. The resulting rice plants accumulated significant amounts of β-carotene in the endosperm compared to non-transgenic plants without β-carotene in the endosperm. 

Metabolic engineering, complemented by genome editing techniques, allows researchers to improve desired experimental results. Thus, the transfer of a transgenic construct including an expression cassette of carotenoid biosynthesis genes (maize *PSY*, Arabidopsis *ORHis*, barley *HGGT*), together with CRISPR/Cas9 sequences to knock out the *β-carotene hydroxylase 2* (*BCH2*) gene, increased the carotenoid content in Arabidopsis seeds by 5.3 times and their stability during ripening and storage. Due to the knockout of the *BCH2* gene, the negative effect of increased carotenoid content on seed storage and germination was reduced, since the pool of hydroxylated β-carotene, which is a precursor in abscisic acid biosynthesis, decreased [146].

We summarized the results of genome editing by CRISPR/Cas9 in various species of higher plants indicating target genes and the type of editing in Table 2.

## 3. Flavonoids

### 3.1. Biosynthesis of Flavonoids

**Figure 3:**
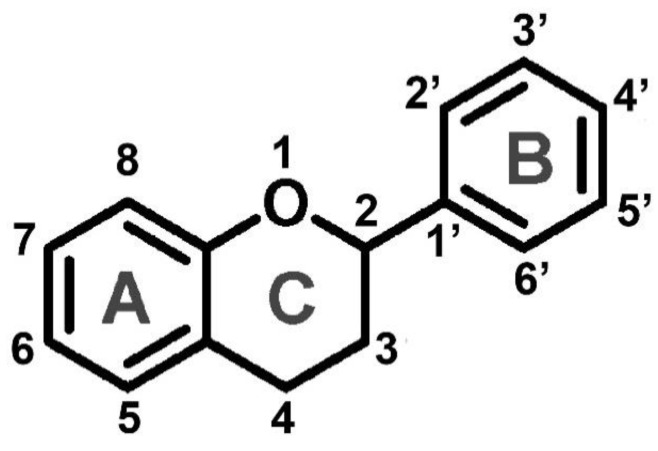
Basic flavonoid skeleton. Benzene rings A and B and heterocycle C are shown in a flavonoid structure.

Flavonoids are a class of water-soluble polyphenolic secondary metabolites containing a 15-carbon phenylpropanoid core, which is modified by rearrangement, alkylation, oxidation, and glycosylation. In the structure of flavonoids, benzene rings A and B, along with heterocycle C, include not only carbon atoms but also oxygen (Figure 3). Flavonoids represent the most numerous classes of natural phenolic compounds. The classification of flavonoids into 12 groups is based on the oxidation state of heterocycle C and the number of hydroxyl or methyl groups on the benzene ring. The four key classes of flavonoids—chalcones, flavanones, dihydroflavonols, and leucoanthocyanidins—also act as intermediate metabolites. They contribute to the synthesis of other flavonoid forms such as flavones, isoflavones, flavonols, flobaphenes, proanthocyanidins, anthocyanins, stilbenes, and aurones.

By functioning as signaling molecules, allelopathic compounds, phytoalexins, detoxifying agents, and antimicrobial protective compounds, flavonoids protect plants from various biotic and abiotic stresses as well as nutritional deficiencies [174]. In addition, flavonoids in plants serve as natural UV filters [175].

**Figure 4:**
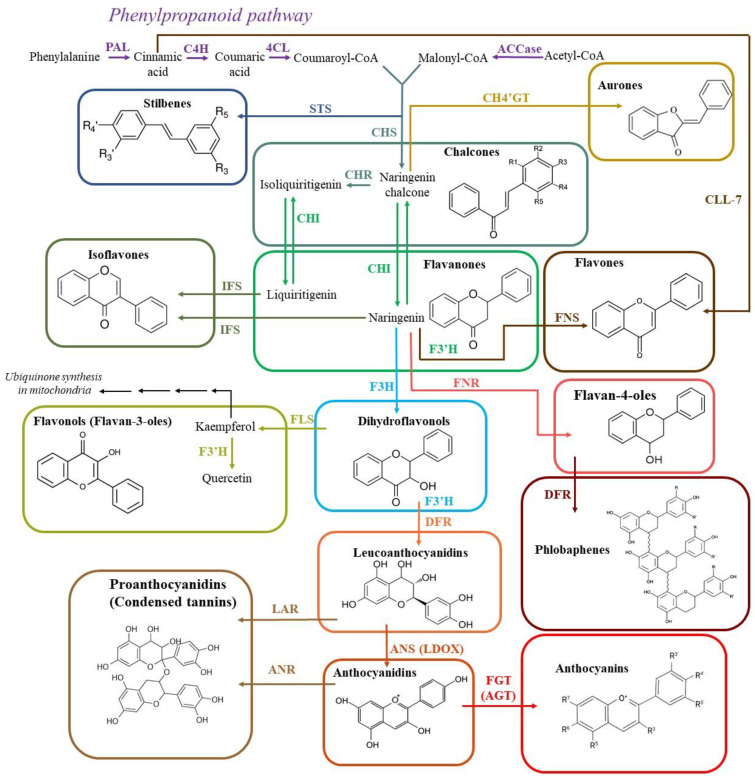
The pathway of flavonoid biosynthesis in plants. Each colored frame represents a different class of flavonoids. The enzyme names are abbreviated as follows: ACCase, acetyl-CoA carboxylase; ANS, anthocyanidin synthase; ANR, anthocyanidin reductase; C4H, cinnamic acid 4-hydroxylase; CHI, chalcone isomerase; CH4′GT, chalcone 4′-O-glucosyltransferase; 4CL, 4-coumarate CoA ligase; CHS, chalcone synthase; CHR, chalcone reductase; CLL-7, cinnamate–CoA ligase; DFR, dihydroflavonol 4-reductase; FGT (AGT), flavonoid glycosyltransferases; FNS, flavone synthase; FNR, flavanone 4-reductase; F3H, flavanone 3-hydroxylase; F3′H, flavanone 3′-hydroxylase; FLS, flavonol synthase; IFS, isoflavone synthase; LAR, leucoanthocyanidin reductase; PAL, phenylalanine ammonia lyase; STS, stilbene synthase.

Biosynthesis of flavonoids (Figure 4) occurs at the cytosolic side of the endoplasmic reticulum, leading to the accumulation of flavonoids in the central vacuole. As with isoprenoids, flavonoid biosynthesis begins with phenylalanine, one of the products of the shikimate pathway (Figure 2). The first three steps of flavonoid biosynthesis from phenylalanine are referred to as the general phenylpropanoid pathway resulting in the formation of 4-coumaroyl-CoA (4-C-CoA) from p-coumaric acid (Figure 4). The initial step, the deamination of phenylalanine to trans-cinnamic acid, is catalyzed by phenylalanine ammonia lyase (PAL) [176]. The information on the genes, which encode the main enzymes for flavonoid biosynthesis in *A. thaliana*, is given in Table 3. PAL exhibits strong inducibility under stress conditions, enhancing the synthesis of all phenolic compounds, including phytoalexins, thereby serving as a primary adaptive response to a number of biotic and abiotic stressors [177]. Subsequent steps of this pathway are catalyzed by cinnamic acid 4-hydroxylase (C4H) and 4-coumarate CoA ligase (4CL). 

The key reaction in flavonoid biosynthesis is the condensation of activated coumaric acid (4-coumaroyl-CoA) with three molecules of activated malonic acid (malonyl-CoA). This reaction is catalyzed by chalcone synthase (CHS), which is the key and the rate-limiting enzyme in the flavonoid biosynthetic pathway. CHS activity leads to the formation of naringenin-chalcone [194]. Total flavonoid level decreased in tomato plants after RNAi-mediated *CHS* gene suppression [195]. Malonyl-CoA and p-coumaroyl-CoA are also used in the synthesis of stilbenes catalyzed by stilbene synthase (STS) [196]. The formation of stilbenes is the first branch of the flavonoid biosynthetic pathway that has only been found in a few plants, such as grapes, pine, sorghum, and peanuts [197,198].

Naringenin-chalcone is the origin for other chalcones after hydroxylation of the ring B. Its formation is also a key branch point for the synthesis of several major classes of flavonoids: flavanones, flavonols, and anthocyanins. Naringenin-chalcone is then exposed to dehydroxylation by chalcone reductase (CHR), yielding isoliquiritigenin [199]. Chalcone isomerases (CHIs) utilize either naringenin-chalcone or isoliquiritigenin to generate naringenin and liquiritigenin, which are another class of flavonoids: flavanones. These flavanones, as well as pentahydroxyflavanone and eriodictyol, are common substrates for the synthetic branches leading to flavones, isoflavones, and phlobaphenes [200,201]. Aurones, yellow plant pigments, which are also synthesized from chalcones [202,203] are found in a relatively small number of plant species, such as snapdragon, sunflower, and coreopsis.

Hydroxylation of naringenin by flavanone 3-hydroxylase (F3H) and flavonoid 3′-hydroxylase (F3′H) produces dihydroflavonols, from which dihydroflavonol reductase (DFR) produces leucoanthocyanidins (Figure 4). The role of the carrot *F3H* gene was tested on model purple calli. Targeted knockout of this *F3H* gene using the CRISPR/Cas9 system led to color loss of these calli (Table 2) [148]. 

With the help of the flavonol synthase (FLS) enzyme, dihydroflavonols are converted into flavonols (Flavan-3-ols) [204] with kaempferol synthesized first, followed by the subsequent formation of other flavonols. The transgenic tobacco mutant containing the *FLS* gene from *Camellia sinensis* showed the accumulation of kaempferol and a decrease in the anthocyanin content in flowers [205]. The other transgenic tobacco mutant, overexpressing *FLS* from *Allium cepa*, possessed enhanced quercetin levels in roots [206]. Kaempferol is also important for biosynthesis of 4-HBA, one of the main precursors of UQ synthesis [47]. *A. thaliana* plants with knocked-out genes encoding kaempferol 3-O-rhamnosyltransferase and kaempferol 3-O-glucosyltransferase accumulated an increased amount of UQ [147]. This was due to the impaired glycosylation of kaempferol by these enzymes to form kaempferol 3-O-glycosides that is a concurrent biochemical pathway for 4-HBA synthesis. This increase in UQ levels is attributed to the absence of glycosylation of kaempferol by the knocked-out enzymes, which normally form kaempferol 3-O-glycosides. These glycosides are part of a parallel biochemical pathway that competes with the synthesis of 4-HBA, a precursor for UQ.

F3′H is a cytochrome P450 monooxygenase responsible for the hydroxylation of the 3′-position on the B-ring of flavonoids. This enzyme can catalyze the conversion of dihydroflavonol dihydrokaempferol to dihydroquercetin or kaempferol to quercetin [184]. A site-specific mutation of the *OsF3′H* gene in rice with black grains led to a significant decrease in flavonoid content and, accordingly, a change in grain color to ocher. The same color with the same decrease in flavonoid content was observed in *DFR* gene knockout plants (Table 2) [149]. The knockout of the *F3′H* gene in *Euphorbia pulcherrima* resulted in a significant decrease in cyanidin levels and a consequent change in bract color from red to yellow-red (Table 2) [150]. 

Flavanones are also the substrates in the flavone, isoflavone, and phlobaphene biosynthetic pathways (Figure 4). F3H competes with flavone synthase (FNS), isoflavone synthase (IFS), and flavanone 4-reductase (FNR), respectively, for these common substrates [207]. FNS is subdivided into two classes: FNSI and FNSII. FNSII comprises NADPH- and oxygen-dependent cytochrome P450-membrane monooxygenases, widely distributed in higher plants [208,209]. FNSI consists of soluble 2-oxoglutarate- and Fe^2+^-dependent dioxygenases, which are predominantly found in Apiaceae [210]. Nevertheless, FNSI was also identified and characterized in maize and Arabidopsis [185]. The biosynthesis of flavones is similar in all higher plants, but in *Scutellaria baicalensis*, which is a traditional medicinal plant in China and is rich in flavones, the additional flavone synthetic pathway was found [211]. In this pathway, cinnamic acid is converted directly by cinnamate–CoA ligase (CLL-7) to cinnamoyl-CoA independently of C4H and 4CL enzyme activity (Figure 4), with subsequent formation of flavones baicalein and norwogonin. 

It has been shown that flavanones (naringenin and eriodictyol) are converted to flavan-4-ols upon inhibition of F3H activity due to the catalytic activity of FNR [212]. Flavan-4-ols are the immediate precursors of one more class of flavonoids, phlobaphenes [213]. Phlobaphenes are only synthesized in maize and other cereals [186,214] and this way is controlled by the MYB (myeloblastosis)-type transcription factor (TF) P [215].

Anthocyanidin synthase (ANS), which is also called leucoanthocyanidin dioxygenase (LDOX), converts leucoanthocyanidins to anthocyanins. It was also shown that this enzyme takes part in the proanthocyanidin biosynthesis pathway, which is important for normal vacuole development in Arabidopsis [216]. Proanthocyanidins (tannins) are synthesized from leucoanthocyanidins and anthocyanidins due to the activity of anthocyanidin reductase (ANR) [13] and leucoanthocyanidin reductase (LAR) [217]. LAR, which is responsible for reduction of leucoanthocyanidin to catechin (trans-flavan-3-ol), has been reported in legumes [218], grapes [219] and *Populus trichocarpa* [220], but it has not been found in Arabidopsis.

Flavonoid 3-O-glucosyltransferase (FGT) is involved in the glycosylation of unstable anthocyanidins to stable anthocyanins [221]. Seven genes encoding this enzyme have been identified in the Arabidopsis genome [188,189,190,191]. Their further modifications (acylation, glycosylation, and methylation) lead to the formation of various anthocyanins [222]. 

Flavonoid biosynthesis is under the control of transcriptional regulator complex MBW, composed of the basic helix–loop–helix (bHLH), MYB, and WD40 (tryptophan-aspartic acid (W-D) dipeptide) proteins. The family of bHLH proteins, which is involved in many essential biological processes, is very common in all eukaryotic organisms [223]. 

The ability of MYB TFs to bind to DNA and interact with other proteins is governed by a conserved MYB domain at their N-terminus [224]. According to the number and position of MYB domain repeats, MYB proteins can be divided into four groups (1R-, R2R3-, 3R-, and 4R-MYB), where the most important TFs are R2R3-MYB (for a review, see [225,226]). In *A. thaliana*, the R2R3-MYB gene family is formed by the genes *MYB11*, *MYB12*, and *MYB111* [227]. In Arabidopsis, the knockout of *MYB12* (*ATMYB12*) resulted in reduced amounts of quercetin and kaempferol in the seedlings, and the flavonoid content was increased in ATMYB12-OX plants. However, the plants with either overexpressed or knocked-out *ATMYB12* gene did not show any significant difference in flavonoid content, and there were no obvious changes in their phenotype compared to wild-type plants. The bHLH TFs have also been shown to be involved in the regulation of multiple physiological and developmental processes [223]. 

### 3.2. Activity of Flavonoids towards ROS

Flavonoids possess antioxidant, antimicrobial, anti-inflammatory, and many other properties (see above). They are widely used in medicine, in industry as dyes, tanning agents, etc. The antioxidant effect of flavonoids is to suppress the formation of ROS by chelation of microelements involved in the formation of free radicals, removal of ROS, and inhibition of enzymes that enhance the formation of free radicals. Flavonoids in plants also possess the ability to absorb UV-solar waves, thus inhibiting the over-production of ROS, and to quench ROS after their formation [228]. 

The antioxidant activity of flavonoids depends on the location of functional groups in their structure. The antioxidant activity is mainly based on the presence of hydroxyl groups (OH), presumably in the B and C rings (Figure 3), while hydroxyl groups in the A ring seem to be less important [229,230,231,232]. Flavonoids, similar to quinones, were shown to efficiently scavenge ROS such as O_2_^•−^ and ^1^O_2_, with the constant rates in the latter case ranging from ~10^6^ M^−1^ s^−1^ to 10^8^ M^−1^ s^−1^ in ethanol predominantly owing to physical quenching [233,234]. The most abundant and largest subgroup of flavonoids in fruits and vegetables are flavones and flavan-3-oles. The latter, along with leucoanthocyanins, are the most reduced flavonoids and, in addition, these compounds are most often present in a reduced form in plant cells. Their hydroxyl groups are responsible for biological activity, especially antioxidant activity. Studies on *A. thaliana* mutants, which were genetically modified to overexpress a number of genes related to flavonoid synthesis and its regulatory transcription factors MYB12/PFG1 and MYB75/PAP1, showed increased resistance to drought [235]. Excessive accumulation of anthocyanins with strong antioxidant activity in vitro reduced the accumulation of ROS in vivo under drought conditions.

Detailed analysis of the antioxidant properties of flavonoids was performed in [232] to understand which ring is the most important for antioxidant activity. In the study, various flavonoids—flavonols, flavanones, flavones, anthocyanidins, hydroxycinnamates, and flavanols—were studied. These compounds have structural similarities but differ in the nature of their B and C rings. The focus was to understand their reactions with 2,2′-azinobis-(3-ethyl benzothiazoline 6-sulfonic acid) diammonium salt radical (ABTS^•+^). ABTS^•+^ is known to be a reactive compound, e.g., the rate constant of ABTS^•+^ with ascorbic acid at neutral pHs is 10^6^ M^−1^ s^−1^ [236]. The study shows that the structure of the B ring, namely the position of hydroxyl group(s), is the main factor determining the antioxidant activity of flavonoids.

The distinctive feature of flavonoids is their ability to function as antioxidants both in the aqueous phase and in lipophilic environments. Therefore, flavonoids neutralize O_2_^•−^ in both water and membrane phases; in both phases, O_2_^•−^ can be produced at a high rate. ^1^O_2_ is mainly generated within PS II (see above) or by any free chlorophyll molecule in chloroplasts to a very small extent. Flavonoids were found to be bound to the chloroplast envelope, so ^1^O_2_, if produced by the free chlorophylls in the vicinity to the envelope, should be accessible to these flavonoids [237]. 

The phenolic hydroxyl groups in flavonoid structure are responsible not only for direct antioxidant activity of flavonoids but also for chelation of metals preventing their interaction with H_2_O_2_ and therefore the generation of highly reactive oxidizer, hydroxyl radical (OH^•^). Besides their direct antioxidant functions, flavonoids also act indirectly by inhibiting ROS-generating enzymes, such as mitochondrial succinoxidase, NADH oxidase, microsomal monooxygenase and others, as well as by upregulating and protecting antioxidant systems [238].

### 3.3. Genetic Approaches for Boosting Flavonoid Production in Plants

Mutagenesis of plants for enhancement of flavonoid production is also a rapidly developing area. There are two main approaches to increase the level of various flavonoids in plants: the regulation of the activity of individual enzymes involved in flavonoid metabolism or the regulation of the activity of TFs, which in vivo are involved in the activation of the entire flavonoid biosynthesis pathway.

#### 3.3.1. Regulation of the Expression of Individual Genes Encoding Key Enzymes in Flavonoid Biosynthesis

Amplification of the expression level of the genes encoding the enzymes of the initial stages of flavonoid biosynthesis sometimes gives ambiguous results. For instance, overexpression of the *CHI* gene (Figure 4, Table 3) in tomato fruits led to a remarkable 78-fold increase in flavonol content [239]. Overexpression of the *CHI* gene from *Petunia hybrida* in potato plants resulted in a slight but significant increase in flavonoid content [240]. 

CHS is responsible for catalyzing the formation of chalcones—a distinct class of flavonoids and a precursor to other flavonoid classes. When the *CHS* gene is overexpressed in potato plants, it frequently results in unexpected outcomes. It often leads to reduction in CHS levels in the plants [240]. Apparently, this effect was observed due to suppression of *CHS* gene expression by its extra copies. However, when the *CHS* gene from barley was overexpressed in potato plants, this effect was not observed, although no significant increase in anthocyanin accumulation was detected either [240].

Flavan-3-ols formation is directly catalyzed by FLS (Figure 4). The effect of the intensification of *FLS* gene expression on flavonoid accumulation and the resistance of resulting plants to various stresses was studied using different plant species. For instance, overexpression of the *FLS* gene from *Euphorbia kansui* Liou in Arabidopsis resulted in an approximately 75% increase in flavonoid content compared to the wild-type plants [241]. The transgenic Arabidopsis plants generated in this study demonstrated enhanced resistance to salinity (200 mM NaCl) and drought induced by 20% PEG. These plants accumulated lower levels of ROS compared to control plants. This was attributed to the antioxidant activity of flavonoids and the increased activity of superoxide dismutase and peroxidase in these plants [241]. Transgenic tobacco plants overexpressing FLS from *Apocynum venetum* also contained a significantly higher amount of flavonoids, approximately 2.8 times more than the wild-type plants [242]. These plants accumulated lower levels of ROS and exhibited increased resistance to salinity (150 mM NaCl) [242]. Arabidopsis plants with overexpression of FLS1 from *Triticum aestivum* were also more resistant to salt stress [243]. However, there are data in the literature that overexpression of FLS does not always lead to an increase in the flavonoid content in plants. For example, in Arabidopsis plants with an overexpressed *FLS1* gene, the flavonoid content did not significantly differ from that in the wild-type plants [244]. It appears that different *FLS* genes perform distinct roles in flavonoid biosynthesis, and selecting the right gene variant is essential.

An increase in the content of flavonoids in the transgenic plants of tea and tobacco was found after overexpression of the *DFR* gene [245], encoding the enzyme, catalyzing the production of leucoanthocyanidins, an alternative class of flavonoids (Figure 4, Table 3). The extract from these plants demonstrated the elevated ability to scavenge stable diphenylpicryl hydrazyl free radicals (70–185%), indicating increased antioxidant activity compared to wild-type plants. Furthermore, these tobacco plants exhibited an increased resistance to biotic stress, particularly against *Spodoptera litura* infestation [245]. The growth of larvae on the transgenic tobacco plants was inhibited by 10–40% compared to wild-type plants, likely due to the reduced ability of the larvae to feed on tobacco with increased flavon-3-ol content. Similar results were obtained in the same study for transgenic tobacco plants overexpressing ANR, an enzyme involved in the synthesis of proanthocyanidins from anthocyanidins, for which leucoanthocyanidins are the precursors (Figure 4). Overexpression of DFR from *P. hybrida* in potatoes resulted in an increase in anthocyanin content. Pelargonidin content increased four-fold and petunidin content increased three-fold [240]. Furthermore, extracts from the tubers of these transgenic potato plants exhibited enhanced antioxidant activity compared to that of wild-type plants [240].

Since anthocyanidins are also the precursors of anthocyanins (Figure 4), the *DFR* gene is often used as a target for genome editing in various plant species. The genome of *Ipomoea nil* contains three tandemly arranged copies (*DFR-A*, *DFR-B*, and *DFR-C*). All these copies are expressed, but *DFR-B* is the dominant one responsible for stem and flower pigmentation. Targeted knockout of the *InDFR-B* gene of *I. nil* resulted in a change in stem color to green and to the appearance of white flowers without anthocyanins (Table 2) [154]. Editing of the *OsDFR* gene in rice resulted in reduced anthocyanin accumulation and a change of the rice grain color from black to ocher (Table 2) [149]. The tomato *DFR* gene was used for two-stage editing in order to develop the technology of inserting a transgene into a given region of the genome. Initially, the authors used CRISPR/Cas9 to obtain tomato plants with a 1013 bp deletion of the DFR gene. In the second stage, this deletion was corrected by restoring the original gene sequence through the use of donor DNA. Knockout of the *DFR* gene led to green color of hypocotyls and calli of seedlings homozygous for the deletion, which were able to regenerate in vitro. When the integrity of the gene was restored by knocking, the purple color of these plants was also restored due to the accumulation of anthocyanins (Table 2) [152].

FNS catalyzes the final step in the formation of one more flavonoid class, flavones (Figure 4, Table 3). Overexpression of FNS from the Antarctic moss *Pohlia nutans* in Arabidopsis resulted in an increased flavone content in the transgenic plants [246]. These transgenic plants demonstrated greater resistance to drought compared to wild-type plants. For instance, seed germination on MS medium with mannitol (osmotic stress) was approximately 45% higher in the transgenic lines compared to the wild-type plants [246]. Transgenic tobacco plants overexpressing FNS from *Morus notabilis* also accumulated more flavones and exhibited increased resistance to UV-B radiation [210].

Genome editing of gentian plants, which produce blue flowers due to the accumulation of the polyacylated anthocyanin gentiodelphin, helped to identify the functions of three genes of the FGT family: *anthocyanin 5-O-glycosyltransferase* (*Gt5GT*), *anthocyanin 3′-O-glycosyltransferase* (*Gt3′GT*), and *anthocyanin 5/3′-aromatic acyltransferase* (*Gt5/3′AT*). In mutant gentian lines, the effect of gene knockouts on pigment accumulation was distinct for each gene. When the *Gt5GT* gene was knocked out, delphinidin 3G became the primary accumulated pigment. For lines with a *Gt3′GT* knockout, the dominant floral pigment was delphinidin 3G-5CafG. Conversely, plants with a *Gt5/3′AT* gene knockout accumulated two types of pigments: delphinidin 3G-5G-3′G as the primary pigment and delphinidin 3G-5G as the secondary pigment. Therefore, there are two possibilities for modification of delphinidin 3G-5G in gentian flowers: one involves glycosylation by the 3′GT enzyme, and the other involves acylation by 5/3′AT. The flowers of the knockout plants were pale red-violet, dull pink, and lavender, in contrast to the bright blue flowers of wild-type plants [156].

#### 3.3.2. Regulation of Transcription Factor Activity to Enhance Flavonoid Biosynthesis

The regulation of flavonoid biosynthesis, which involves the participations of transcription factors bHLH and the proteins of MYB family is described above. The genes of the R2R3-MYB subfamily of transcription factors are actively used as targets for genome editing to increase the level of flavonoids in plants [247,248]. Overexpression of *AtMYB12* in Arabidopsis simultaneously increases the expression of *AtCHS*, *AtCHI*, *AtF3H*, and *AtFLS* (Table 3) [247,248]. The resulting transgenic Arabidopsis plants exhibited enhanced resistance to drought and salinity (25% PEG6000 or 200 mM NaCl for 2 weeks) [248]. The levels of H_2_O_2_ and malondialdehyde (MDA) in the transgenic plants were 40–60% lower compared to the wild-type plants, while the activities of SOD and peroxidase were 30–40% higher [248].

Transgenic tobacco plants overexpressing *AtMYB12* also demonstrated increased resistance to biotic stress caused by insect pests, such as *Spodoptera litura* and *Helicoverpa armigera* [10]. The enhanced resistance of transgenic plants is likely associated with the accumulation of the flavonoid rutin, which is toxic to insects.

PAP1 (Production of Anthocyanin Pigment 1) is another transcription factor, a typical representative of the R2R3-MYB transcription factors. Overexpression of *AtPAP1* in tobacco led to increased accumulation of flavonoids in all parts of the plant [249]. In these transgenic tobacco plants, the expression of genes *PALs*, *CHS*, *CHI*, *F3H*, *F3′H*, *ANS*, and *DFR* (Table 3, Figure 4) was significantly higher compared to control tobacco plants. Furthermore, these transgenic plants also demonstrated increased resistance to insect pests, such as *S. litura* [249].

Arabidopsis double mutant *WOX1*, which simultaneously overexpresses both MYB12 and PAP1 TFs, contained up to 20 times more anthocyanins in all parts of the plants, although the overall flavonoid content only increased two-fold [235]. The *WOX1* plants accumulated more anthocyanins and total flavonoids than the plants that individually overexpress either MYB12 or PAP1. These double mutants demonstrated greater radical scavenging activity and increased resistance to oxidative stress induced by methyl viologen, approximately three-fold compared to the wild-type plants [235]. Additionally, the double mutants showed enhanced drought resistance (no watering for 20 days) compared to the wild-type plants.

Simultaneous expression of two maize transcription factors, MYB type C1 and MYC type LC, under the control of the fruit-specific tomato E8 promoter led to an increase in the content of kaempferol (approximately 60-fold), naringenin (2-fold), and quercetin (3-fold) in tomato fruits [250]. In *C1/LC* transgenic tomato plants, the expression of *CHS*, *F3H*, and *DFR* increased by over 100-fold, while the expression of *FLS* and *ANS* was also higher in transgenic plants, by 5 to 15 times compared to the wild-type plants [250].

The tomato genome contains four homologous R2R3-MYB transcription factors: SlAN2, SlANT1, SlANT1-like, and SlAN2-like/Aft. Dark purple tomato plants were obtained by inserting the 35S CaMV promoter into the promoter region of the *SlANT1* gene using the TALENs and CRISPR/Cas9 systems, and the bean yellow dwarf virus (BeYDV) vector for producing donor DNA. This strategy increased the expression level of the TFs, which resulted in an increased expression level of genes responsible for flavonoid synthesis [158]. Insertion of the CaMV 35S into the promoter region of the *SlANT1* gene using CRISPR/LbCpf1-based HDR resulted in pronounced plant pigmentation and allowed visual selection of edited plants [159]. Tomato *SlAN2-like* mutants generated by CRISPR/Cas9 accumulated less amounts of flavonoids and were dysregulated in the expression of many flavonoid biosynthesis genes [160]. The *SlAN2* mutation introduced using CRISPR/Cas9 into purple tomatoes cv. “Indigo Rose” led to a decrease in the content of flavonoids only in the vegetative parts of plants and was accompanied by a number of morphological changes (reduction in plant height, decrease in fruit size). In the fruits of the edited plants, the flavonoid content was the same as in the unedited plants [161]. In carrots, the knockout of the *DcMYB113-like* gene in the purple variety resulted in depigmented regenerants [54].

The R2R3-MYB transcription factor PtrMYB57 is a repressor of anthocyanin and proanthocyanidin biosynthesis. In poplar, the CRISPR/Cas9 mutant *PtrMYB57* was characterized by high levels of anthocyanin and proanthocyanidin in leaves compared to wild-type plants [162]. The R2R3-MYB transcription factor FtMYB45 suppresses flavonoid biosynthesis in Tartary buckwheat (*Fagopyrum tataricum*). Knockout of this TF resulted in increased content of rutin, catechin, and other flavonoids in hairy root mutants [163].

In the basic helix–loop–helix (bHLH) group of TFs, the *Transparent Testa* gene plays an important role in the accumulation of flavonoids. CRISPR/Cas9 BnTT8 mutants in *Brassica napus* possessed yellow seed color associated with a block in tissue-specific proanthocyanidin deposition in the seed coat, as well as increased protein and lipid content. Transcriptome analysis showed that targeted mutations resulted in suppressed expression of phenylpropanoid/flavonoids biosynthetic genes [167]. Targeted mutagenesis of homologous *TT8* genes in tobacco (*NtAn1a* and *NtAn1b*) also resulted in seeds with a yellow seed coat phenotype and increased lipid and protein accumulation [168].

In the MYB-bHLH-WD40 (MBW) complex, the repeat protein WD40 is involved in transcriptional regulation of the flavonoid metabolic pathway in many plant species. The *Transparent Testa Glabra1* (*TTG1*) locus related to WD40 was knocked out using CRISPR/Cas9 gene editing technology in *A. thaliana* plants with different ploidy levels [165] and in rice plants [166]. Mutations in the gene resulted in a decrease in the synthesis of flavonoids in plants that led to light coloration of seeds and disturbances in the formation of trichomes.

The basic region/leucine zipper (bZIP) TF gene family also plays a key role in the regulation of flavonoid biosynthesis in many plant species. Knockout of the *VvbZIP36* gene in grapevine (*Vitis vinifera*) using CRISPR/Cas9 led to the accumulation of flavonoids and a number of related metabolites (naringenin chalcone, naringenin, dihydroflavonols, and cyanidin-3-O-glucoside), which was accompanied by the appearance of red pigmentation on the leaves. Editing revealed that VvbZIP36 is a negative regulator of flavonoid biosynthesis [164].

Thus, obtaining targeted mutations in various transcription factor genes makes it possible to identify their functions in regulating the expression of flavonoid biosynthesis genes (activation or repression) and clarify their role in other processes related to plant development. Other TFs, such as PAP1, MYB1, MYB2, MYB10, DcMYB6, and the Lateral Organ Boundary Domain (LBD) TF family are known to increase the accumulation of flavonoids. These TFs are potential candidates for genome editing [251,252]. An example is *FaMYB10-2*, one of three MYB10 homologues responsible for fruit color in strawberries. It is known that the insertion of a transposon into the promoter region of this gene can alter the biosynthesis of flavonoids. Depending on the location, this insertion can either enhance the biosynthesis of flavonoids in fruits or lead to its inhibition and the appearance of white fruits [253]. Using genome editing, it becomes possible to obtain various types of mutations with desired change in the phenotype of cultivar strawberry fruits.

Uridine diphosphate-dependent glucosyltransferases (UGTs) are responsible for the transfer of monosaccharide residues to their acceptor molecules in plants. Two enzymes, encoded by the *UGT79B2* and *UGT79B3* genes, were identified in Arabidopsis. These enzymes participate in modifications of flavonoids by adding UDP-rhamnose to cyanidin and 3-O-glucoside-cyanidin. The double mutants *ugt79b2/b3* generated by the CRISPR/Cas9 system were characterized by reduced levels of flavonoids in plants and increased susceptibility to abiotic stress. Thus, UGT79B2 and UGT79B3 are flavonoid rhamnosyltransferases, and they mediate abiotic stress tolerance by modulating flavonoid accumulation [155].

## 4. Ascorbate and Glutathione

### 4.1. Biosynthesis of Ascorbate

Ascorbate, also known as vitamin C or L-ascorbic acid (Asc), is an important biologically active substance. Despite its importance, the pathway of its biosynthesis in plant cells was described only in 2007 for *A. thaliana* and many aspects concerning the regulation of the Asc biosynthetic pathway still need clarification. Plants synthesize Asc by four alternative routes: D-mannose/L-galactose (Smirnoff–Wheeler) [254], L-Gulose, Myo-inositol, and D-Galacturonic pathways [255,256,257]. The Smirnoff–Wheeler pathway is often referred to as the primary or main pathway of Asc biosynthesis in plants [258]. However, in plant species which produce fruits with high level of vitamin C, the other known pathways for Asc biosynthesis can also be predominant. For example, the L-galactose pathway is the main one in peaches and kiwis, while the D-galacturonic pathway is dominant in grapes and strawberries [259]. Additionally, in a number of plants, such as citrus or tomato, the predominant biosynthetic pathway can shift as the fruits ripen.

**Figure 5:**
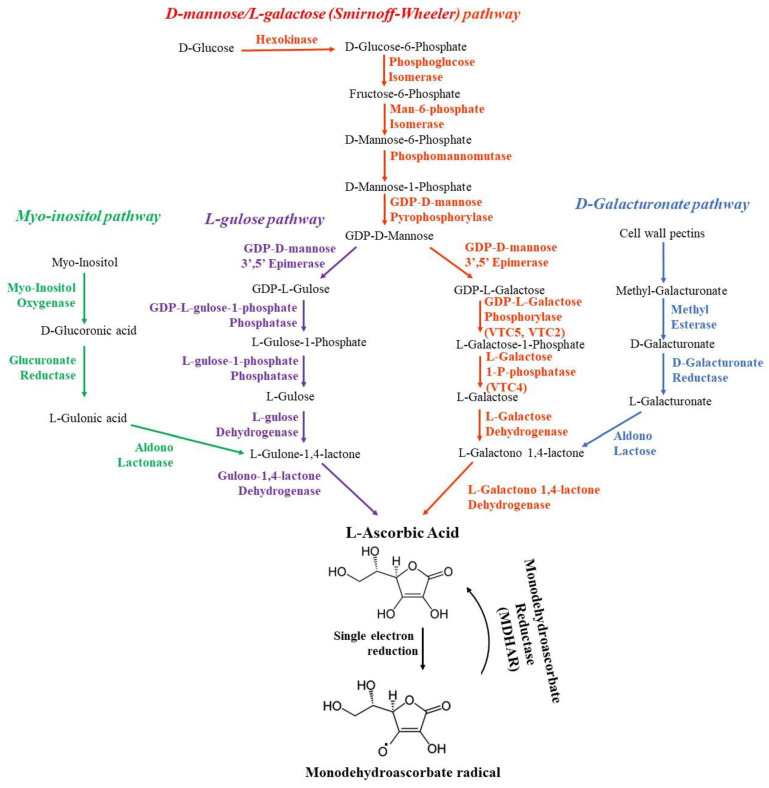
The pathways of L-ascorbic acid biosynthesis in plants. The enzymes catalyzing the reactions of Smirnoff–Wheeler, L-gulose, D-Galacturonate, and Myo-inositol pathways are red, purple, blue, and green, respectively.

Most enzymes of the D-mannose/L-galactose pathway are localized in the cytosol. This pathway starts from the conversion of D-glucose into L-galactose through eight stages, which are necessary only to change the spatial position of the hydrogen and hydroxyl groups at the fourth carbon atom in the structure of these carbohydrates (Figure 5). The first step is the transformation of D-glucose to D-glucose-6-phosphate, followed by its transformation into D-fructose-6-phosphate by phosphomannose isomerase (PMI). D-fructose-6-phosphate is further converted into D-mannose-6-phosphate also by phosphomannose isomerase. D-Mannose-6-phosphate is converted into D-Mannose-1-phosphate by phosphomannose mutase (PMM). D-Mannose-1-phosphate is then transformed to GDP-D-Mannose by GDP-D-mannose pyrophosphorylase (GMP). Studies have indicated a correlation between *PMI1* gene expression in Arabidopsis and ascorbate levels [260]. The same authors have shown that the knockdown of *PMI1* has led to decreased Asc levels. In transgenic tobacco overexpressing the *PMM*, *GDP*, and *GMP* genes derived from *Malpighia glabra* (a plant known for its remarkably high vitamin C content), the Asc levels were found to be two-fold higher than in wild-type plants [261,262,263]. The information about the genes encoding the main enzymes for Asc biosynthesis in *A. thaliana* is in Table 4.

Secondary metabolic reactions that facilitate the transformation of GDP-L-galactose into Asc (Figure 5) are active mainly in mature plants. The rate-limiting step in this metabolic pathway of vitamin C synthesis is the reaction that directly produces Asc, controlled by the enzyme GDP-L-galactose phosphorylase. The enzyme GDP-D-mannose-3′,5′-epimerase (GME) controls the mutual transformation of GDP-D-mannose and GDP-L-galactose. In young, actively growing plants, most products from this reaction contribute to primary metabolic reactions, specifically, the biosynthesis of cell wall polysaccharides [278,279]. This suggests that the initial stages of this metabolic pathway are mainly associated with the growth of plant organs. This implies that the first specific step for L-Ascorbic acid biosynthesis in the Smirnoff–Wheeler pathway is the conversion of GDP-L-galactose into L-Galactose-1-phosphate, a reaction catalyzed by GDP-L-galactose-phosphorylase (GGP) [280]. In Arabidopsis, this enzyme is encoded by two genes, *VTC2* and *VTC5* (Table 4) [281]. The plants with knockout of these genes had 20% and 80% of the vitamin C content of wild-type plants, respectively [270]. The double *vtc2* and *vtc5* mutants were unable to grow beyond the cotyledon expansion. Adding galactose or Asc to the seedlings compensated for the absence of both of these enzymes. These data suggest that, at least in Arabidopsis, the D-mannose/L-galactose pathway is the main source of vitamin C.

*VTC4* in Arabidopsis encodes galactose-1-phosphate phosphatase (GPP), which converts L-galactose-1-phosphate to L-galactose [271,282]. Knockouts of the *VTC4* gene in Arabidopsis resulted in only a partial decrease in GPP activity and vitamin C content [271,272]. This unexpected retention of activity can be attributed to the fact that, in Arabidopsis, this reaction is also catalyzed by purple acid phosphatase AtPAP15 (Table 4) [273]. The conversion of L-galactose to L-galacton-1,4-lactone is catalyzed by L-galactose dehydrogenase (GDH), which is an NAD-dependent enzyme [254]. Overexpression of GDH in Arabidopsis did not change the content of vitamin C, and in antisense plants, the content of vitamin C decreased only under bright light [283], which suggests that it is not a crucial stage of Asc biosynthesis. The last step of the L-galactose pathway is the oxidation of L-galactono-1,4-lactone by L-galactono-1,4-lactone dehydrogenase (GLDH) into Asc (Figure 5). This step takes place in mitochondria, and is coupled with the cytochrome *c* pathway that is present there [284]. GLDH was identified as one of the proteins in mitochondrial complex I [285]. It acts as an essential plant-specific factor for complex I assembly [286]. Thus, stresses that disrupt electron flow have significant effects on ascorbate biosynthesis [284].

The second important pathway for Asc biosynthesis in plants is the D-galacturonate pathway, which involves methyl-D-galacturonate from pectin of cell walls (Figure 5). In the first step of this pathway, methyl esterase catalyzes the conversion of methyl-D-galacturonate into D-galacturonate, which is then transformed into L-galacturonate by D-galacturonate reductase. It has been shown that D-galacturonate methyl ester supplied exogenously to cell cultures, including those of Arabidopsis, caused an increase in ascorbate level [287,288]. The *GalUR* gene, which expression correlates with the increase in Asc during fruit ripening, was cloned, and the recombinant enzyme was shown to have NADPH-dependent D-GalUA reductase activity [257]. Its role in ascorbate biosynthesis was confirmed by overexpression in Arabidopsis, which resulted in a several-fold increase in foliar ascorbate. However, there is no evidence for this pathway operating under normal conditions; it takes place only under conditions of cell wall breakdown [289].

Exogenous L-gulose addition also increased the Asc content of Arabidopsis cell cultures but much less effectively than the addition of methyl galacturonate [287]. In the L-gulose pathway of Asc biosynthesis, the precursor of L-gulose is GDP-D-mannose, a compound also found in the Smirnoff–Wheeler pathway (Figure 5). The first step of the L-gulose pathway is the conversion of GDP-D-mannose into GDP-L-gulose by GDP-D-mannose-3′,5′-epimerase (GME), followed by the formation of L-gulose-1-phosphate by GDP-L-gulose-1-phosphate phosphatase. In the last step of this pathway, AsA is formed from L-gulono-1,4-lactone by L-gulono-1,4-lactone dehydrogenase in mitochondria.

The myo-inositol pathway includes conversion of myo-inositol into L-gulonic acid, with its subsequent lactonization to L-gulono-1,4-lactone by aldono lactonase (Figure 5). Then L-gulono-1,4-lactone dehydrogenase converts L-gulono-1,4-lactone into Asc, a step also seen in the gulose and the Smirnoff–Wheeler pathways [264,290]. It was also shown that the transformation of lettuce and tobacco by constitutive expression of the rat cDNA encoding L-gulono-1,4-lactone oxidase resulted in a four- to seven-fold increase in the content of vitamin C in the leaves of these plants [291].

Experimental data demonstrating the significance of the myo-inositol metabolic pathway for Asc synthesis are contradictory. The main enzyme in this pathway is myo-inositol oxygenase (MIOX). Overexpression of the gene encoding this enzyme (*At4g26260*) in Arabidopsis was reported to increase the content of foliar ascorbate [256]. Later, Endres and Tenhaken [292] showed a decrease in myo-inositol with no changes in the Asc level in the same transgenic plants. Nevertheless, in a later study, Arabidopsis lines overexpressing MIOX exhibited an elevated level of foliar Asc, enhanced growth rate, higher biomass accumulation, and increased tolerance to abiotic stresses [293]. MIOX4 overexpressing lines also had an increased level of auxin, showing an increase in the expression of genes involved in auxin metabolism, as well as an increased PS II efficiency and an increased proton motive force [294].

Asc synthesis in higher plants is modulated on both transcriptional and post-transcriptional levels. However, our understanding of the regulatory mechanisms both within and between the Asc synthesis pathways remains limited. Ascorbic Acid Mannose Pathway Regulator 1 (AMR1) is a negative regulator of Asc and mannose pathways. AMR1 negatively affected the expression of the genes encoding GMP, GME, GGP, GPP, GDH, and GLDH [276]. As the light intensity increased, the content of the *AMR1* gene transcripts decreased. With aging of plant leaves, the accumulation of *AMR1* gene transcripts was observed, which coincided with a decrease in Asc level. *AMR1* knockout mutants showed higher accumulation of Asc levels and were more tolerant to oxidative stresses. Ethylene response factor ERF98, which is induced by ethylene, salt, and H_2_O_2_, transcriptionally activates Asc synthesis [276]. Arabidopsis mutant plants with *AtERF98* gene knockout and knockdown exhibited decreased Asc levels, while mutants with overexpressed *AtERF98* showed increased levels.

### 4.2. Biosynthesis of Glutathione

**Figure 6:**
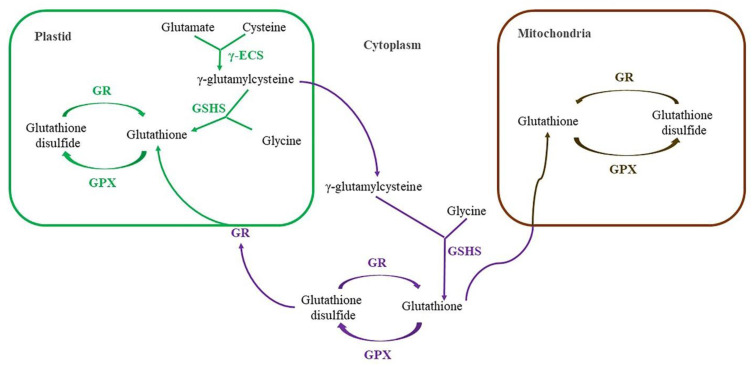
The pathways of glutathione biosynthesis in plants. The enzyme names are abbreviated as follows: γ-ECS, γ-glutamylcysteine synthetase; GSHS, glutathione synthetase; GR, glutathione reductase; GPX, glutathione peroxidase. The cytoplasmic stages are marked in purple; the plastid stages are marked in green; the mitochondrial stages are marked in brown.

The glutathione molecule is a tripeptide consisting of three amino acids: glutamate, cysteine, and glycine. Glutathione synthesis takes place in chloroplasts and cytosol [295,296]. Once synthesized, glutathione can be found in various cellular compartments, including mitochondria. Glutathione is primarily synthesized in chloroplasts, since both of the enzymes involved in this pathway, γ-glutamylcysteine synthetase (γ-ECS) and glutathione synthetase (GSHS), are located in this compartment. The biosynthesis of glutathione consists of two steps (Figure 6). In the first step, γ-ECS catalyzes the reaction between the γ-carboxyl group of glutamate and α-amino group of cysteine, resulting in the formation of γ-glutamylcysteine, which is partially exported to the cytosol. In the second step, GSHS, which is located both in the cytosol and plastids, forms glutathione molecule by amide bond formation between the α-carboxyl group of the cysteine moiety in γ-glutamylcysteine and the α-amino group of glycine [297,298]. After that, glutathione is transported into the mitochondria or reimported into the plastids [299].

The activities of various enzymes affect the reduced/oxidized glutathione ratio, which is also significantly affected by various stresses. The rate of glutathione synthesis is controlled by several factors with the most important one being the feedback inhibition of γ-ECS by glutathione due to its binding to the glutamate site of the enzyme [300]. The information about the genes encoding the main enzymes for glutathione biosynthesis in *A. thaliana* is given in Table 4.

### 4.3. Activity of Ascorbate and Glutathione towards ROS

Asc and glutathione are the main participants in the Foyer–Halliwell–Asada metabolic pathway or the water–water cycle. This cycle serves to detoxify H_2_O_2_ with involvement of Asc, glutathione, and NADPH [301,302,303]. The water–water cycle is initiated when electrons are transferred from photosynthetic electron transport chain to oxygen, forming O_2_^•−^ (see above) [304]. O_2_^•−^ further undergoes dismutation reaction catalyzed by SOD or O_2_^•−^ can be reduced by PQH_2_ (see above), to produce H_2_O_2_, which in turn is reduced by Asc with the involvement of ascorbate peroxidase (APX), generating H_2_O and monodehydroascorbate radical (MDHA) (Figure 5). If MDHA is not reduced by the components of photosynthetic electron-transport chain, it dismutates to form Asc and dehydroascorbate (DHA). DHA is reduced to Asc by glutathione, and the oxidized glutathione is further reduced by NADPH with the involvement of glutathione reductase [301]. Since glutathione, ascorbate, and NADPH are present in plant cells at high concentrations, it is assumed that this cycle plays a significant role in H_2_O_2_ detoxification [258].

Two isoforms of APX are present in chloroplasts, the thylakoid-bound (tAPX) and the soluble stromal (sAPX) [305]. Compared to catalase (which is located in peroxisomes and glyoxysomes in higher plants) with a Km value for H_2_O_2_ of 20–25 mM [306,307], APX, having a lower Km value of 80 μM, can maintain a much lower concentration of H_2_O_2_ in chloroplasts. This is important given the inhibitory effect of H_2_O_2_ on the Calvin–Benson–Bassham cycle enzymes, with a half-inhibitory concentration as low as 10 µM [308,309].

In addition to the antioxidant activity against H_2_O_2_, both ascorbate and glutathione can scavenge O_2_^•−^. The rate constant of the reaction with O_2_^•−^ at neutral pH values is ~10^5^ M^−1^ s^−1^ for ascorbate [310] and ~10^3^ M^−1^ s^−1^ for reduced glutathione [311]. Both reactions lead to the formation of H_2_O_2_ in the chloroplast stroma; however, they are much less efficient than the dismutation reaction of O_2_^•−^ catalyzed by SOD (the rate constant is ~10^9^ M^−1^ s^−1^). Furthermore, ferredoxin, which is the electron acceptor from PSI, in its reduced form is also known to reduce O_2_^•−^ to H_2_O_2_ [312]. However, in vivo, the steady-state concentration of the reduced ferredoxin is low. Given that the addition of SOD effectively inhibits this reaction, the rate constant of the reaction of reduced ferredoxin with O_2_^•−^ is likely not very high [312].

Asc efficiently quenches ^1^O_2_ by chemical mechanism producing DHA and H_2_O_2_ with the rate constant of this reaction estimated to be 3 × 10^8^ M^−1^ s^−1^ [313], while the physical quenching rate is rather low [104]. Reduced glutathione and other thiols predominantly exhibit a chemical quenching mechanism of ^1^O_2_; the rate constant for glutathione is estimated to be 2.4 × 10^6^ M^−1^ s^−1^ [314].

Asc and glutathione are involved not only in the water–water cycle operation and direct scavenging of ROS to minimize the consequences of the oxidative stress in plant cells, but also in regeneration and stabilization of several cell components. For example, Asc plays a role in the regeneration of α-tocopherol from its radicals [11], which are produced during the detoxification of lipid peroxide radicals. Glutathione is also the substrate for phospholipid hydroperoxide glutathione peroxidase, which is one of the glutathione peroxidase (GPX) isoforms. Under stress conditions, this peroxidase is upregulated to protect against the accumulation of lipid hydroperoxides in various cell compartments [315].

Glutathione regulates multiple metabolic functions. It is involved in detoxification, redox homeostasis, and acts as an antioxidant. It interacts with proteins in several ways, affecting their structure and activity through changes in the thiol–disulfide balance and preventing oxidative denaturation under stress conditions by protecting their thiol groups. Glutathione protects membranes by maintaining the reduced state of antioxidants such as α-tocopherol and zeaxanthin. The mechanism of glutathione detoxification of various toxic compounds is based on the conjugation of the xenobiotics with glutathione, leading to the formation of less reactive products. This conjugation reaction can occur spontaneously, but in biological systems, it is presumably facilitated by glutathione S-transferase (GST) (for review, see [316]). Detoxified xenobiotics are then transported to vacuoles for their subsequent sequestration.

### 4.4. The Approaches for Boosting Ascorbate and Glutathione Production

Describing the discovery of enzymes involved in Asc biosynthesis, we have already reported examples of the successful creation of mutants with its increased synthesis. One more approach to obtain an increased production of the metabolites of interest in plants is to regulate the target gene expression at the translational level. For this purpose, the targeted mutations of nucleotide sequences are introduced in the region of upstream open reading frames (uORFs) of the genes of interest. UORFs are cis elements located in the 5′ untranslated regions (UTR) of mRNA; they promote ribosome stalling and dissociation during mRNA translation, thus acting as translation repressors. Introducing mutations into the uORF regions can eliminate this suppressive effect, leading to an increased expression of the gene [317]. This strategy was successfully tested on the uORF of the *LsGGP2* gene, encoding GDP-L-galactose phosphorylase, which is a key enzyme in the biosynthesis of Asc through the Smirnoff–Wheeler pathway. Editing of the uORF led to an increase in Asc content in lettuce (*Lactuca sativa* L.) leaves by approximately 150% and an increased resistance to oxidative stress (Table 2) [173]. A similar study was done with tomato plants. The mutations in the uORF region of the *SlGGP1* gene in two independent CRISPR-edited lines led to an increase in the Asc level in fruits (Table 2) [169]. In mutant tomatoes edited in the uORF-*GGP1* (single nucleotide transversion from C to T) region, the amount of Asc increased by 2–5 times compared to the wild-type plants. However, these mutations led to impaired flower fertility, resulting in the production of homozygous mutants with the fruits having a small number of non-viable seeds or being completely seedless [169].

Another approach to increase the Asc level, which was confirmed experimentally, was the editing of the transcription factors of genes encoding proteins involved in its synthesis. The EIL2 protein controls carotenoid metabolism and Asc biosynthesis in tomatoes (*Solanum lycopersicum*). As previously described, the fruits of the CRISPR/Cas9 *eil2* mutants had changes in fruit color and also showed an increase in ascorbic acid content. The authors showed that *SlEIL2* repressed the expression of the gene *L-GALACTOSE-1-PHOSPHATASE 3* (*SlGPP3*) and *MYO-INOSITOL OXYGENASE 1* (*SlMIOX1*) at the transcriptional level, resulting in a 1.6-fold increase in Asc synthesis through the L-galactose and myo-inositol pathways in the mutants (Table 2) [67].

To increase the level of Asc in plants, genes that regulate its metabolism were proposed as candidates for genome editing. These are the *ascorbic acid mannose pathway regulator 1* (*AMR1*), *COP9 Signalosome Subunit 5B* (*CSN5B*) and *8* (*CSN8*), and *NBS-LRR 33* (*NL33*). This strategy is supported by data from insertional mutagenesis and RNA interference, demonstrating that reduced expression of these genes led to a higher concentration of Asc and enhanced plant stress resistance [318].

Plants with elevated Asc content were obtained by overexpressing monodehydroascorbate reductase (MDHAR), an enzyme that maintains the reduced Asc pool (Figure 5). Tobacco plants overexpressing the *MDHAR* gene from acerola accumulated approximately 1.6 to 2 times more Asc and exhibited greater resistance to salt stress [319]. The content of MDA in the transgenic plants subjected to stress was about two times lower compared to wild-type plants [319]. The co-overexpression of MDHAR and DHAR from *Brassica rapa* in Arabidopsis resulted in an increased glutathione content and an enhanced resistance to freezing (16 h at −5 °C) [320].

Regulating the activity of GR, GST, and GPX, the enzymes directly affecting the levels of reduced/oxidized glutathione ratio, is an effective approach to increase the content of glutathione and the resistance of plants. Overexpression of bacterial GR in chloroplasts of a poplar hybrid (*Populus tremula* × *Populus alba*) led to a 100- to 500-fold increase in GR activity compared to wild-type plants [321]. This resulted in a two-fold increase in the total glutathione content in the leaves and an increase in the reduced fraction of glutathione. The engineered plants exhibited greater resistance to photoinhibitory conditions (1000 µmol quanta/m^2^ s, 5 °C) and oxidative stress induced by leaf incubation in the presence of MV [321].

Overexpression of tomato GR in tobacco plants also led to a 1.9- to 2.3-fold increase in GR activity compared to the wild-type plants [322]. The resulting transgenic plants exhibited higher germination rates and increased root length compared to the wild-type under normal conditions. Moreover, these plants demonstrated better growth under salinity conditions (100 mM NaCl), and lower hydrogen peroxide accumulation [322]. Arabidopsis plants overexpressing *AtGR1* had a higher glutathione level and a higher reduced/oxidized glutathione ratio. Such transgenic plants exhibited greater resistance to the toxic effects of aluminum: H_2_O_2_ production in transgenic plants was 26% lower compared to wild-type plants [323].

Noteworthy results were reported in the study by Raja et al. In this research, tomato plants were engineered with a cassette of genes encoding enzymes of the ascorbate-glutathione cycle. Genes, which were used in this study, included *MDHAR*, *DHAR*, *GR*, *APX*, and *SOD* from *Pennisetum glaucum*, under the control of stress-inducible promoters [324]. These transgenic tomato plants accumulated approximately 50% more ascorbate and 90% more DHA compared to the wild-type plants and demonstrated enhanced resistance to salinity (200 mM NaCl) and drought stress [324]. Furthermore, these engineered plants exhibited increased resistance to mercury (Hg) toxicity, accumulating significantly lower levels of H_2_O_2_ and maintaining higher photosynthetic activity compared to wild-type plants [325]. Notably, the transgenic tomato plants accumulated 20% less Hg in their leaves but 40% more in their roots than the wild-type plants [325].

Similar to the case with flavonoids and ascorbate, a promising approach for glutathione level increase involves the regulation of transcription factor activity. For instance, it has been demonstrated that overexpressing the transcription factor NAC2 from *Solanum lycopersicum* L. in Arabidopsis plants leads to increased expression of enzymes involved in glutathione biosynthesis (γ-ESC, GS, and GR) under abiotic stress conditions [326]. These engineered plants accumulated fewer ROS, exhibited improved growth, and demonstrated better water retention in tissues during drought and salinity stress compared to the wild-type plants [326].

As mentioned above, glutathione together with GST is involved in binding heavy metals. Thus, transgenic plants with increased glutathione content can be used to remediate soil contaminated with heavy metals. For instance, Indian mustard plants with overexpression of glutathione synthetase from *E. coli* and elevated glutathione levels accumulated three times more cadmium in their shoots than wild-type plants [327]. Rice plants overexpressing the *GST* gene *OsGSTU5* contained higher levels of glutathione compared to wild-type plants. In the presence of cadmium in the soil, these transgenic plants accumulated 1.5 times more cadmium than the wild-type plants [328].

Overexpression of *GST* from *Suaeda salsa* (halophytic plant adapted to saline-alkali soils) in Arabidopsis under the control of the 35S promoter led to a significant increase in the expression of GST and GPX [329]. The resulting plants also showed enhanced tolerance to salinity stress (100 mM NaCl). Under saline conditions, transgenic plants did not exhibit an increase in accumulation of MDA, whereas wild-type plants experienced a substantial increase in MDA levels under salinity stress compared to control conditions [329].

Overexpression of tobacco GST led to approximately a 2-fold increase in GST activity and an approximately 1.8-fold increase in the total glutathione content [330]. Constitutive expression of *RcGPX* from *Rhodiola crenulatea* in *Salvia miltiorrhiza* under the control of the 35S promoter also led to an increase in the total glutathione content and the increase in activity of the enzymes GR, APX, and GPX [331]. The resulting plants exhibited greater resistance to a two-week drought compared to the wild-type plants [331]. Tobacco plants expressing *GPX* from Chlamydomonas exhibited increased resistance to oxidative stress induced by methyl viologen, photoinhibitory stress (1000 µmol quanta/m^2^ s, 4 °C), and salt stress (250 mM NaCl) [332]. In Arabidopsis, there are eight isoforms of glutathione peroxidase-like enzymes. Overexpression of one of them, *AtGPXL5*, in Arabidopsis led to an increase in glutathione content. The resulting transgenic plants were more resistant to the effects of 100 mM NaCl compared to the wild-type plants [333].

Analysis of the gene encoding GST, carried out using CRISPR/Cas9 mutant gentian lines, showed that the functioning of this enzyme is associated not only with glutathione, but also with the transport of flavonoids and their accumulation in flowers and leaves [334]. In octoploid strawberries, it was confirmed that the *Reduced Anthocyanins in Petiole* (*RAP*) gene, which encodes GST, plays a crucial role in binding and transporting flavonoids into fruits and leaves. In the initial generation (T0), when six copies of the *RAP* gene were simultaneously knocked out in the strawberry genome, it resulted in a green stem and white fruit phenotype [172].

In gene editing, knocking out the genes which encode enzymes of biosynthesis of colored antioxidants is also employed as a morphological selective marker. For example, tomato plants with male sterility were created by simultaneously knocking out the *male sterile 1035* (*Ms1035*) and *GST* genes. This allowed the creation of a male-sterile tomato line that was selected for the green color of the hypocotyl [170]. Double knockout mutants for both the *GST* gene and the *Ms10* locus, which mediates male sterility and is closely linked to *GST*, had a green hypocotyl and were easily scanned at the seedling stage [171]. In the other study, the authors used a double knockout mutant for the *TM6* (male sterile locus) gene and the gene encoding DFR, one of the enzymes of flavonoid synthesis (see above). These mutations were also inherited in a linked manner, and selected for a green, non-pigmented hypocotyl that indicated the desired mutation variants [153]. 

To increase transcriptional level of PAP1, dCas9 activation system with addition of p65 transactivating subunit of the TF nuclear factor (NF)-kappa B and a heat shock factor 1 (HSF) activation domain, was tested. Editing led to an increase in the expression level of the *PAP1* gene by two to three times and stained *A. thaliana* leaves purple, which confirmed the success of the generated dCas9 construct with modified p65-HSF as a transcription activator [157].

## 5. Conclusions

The versatility of oxidative stress occurrence in response to environmental conditions, like extreme temperatures, drought, soil salinity, pests, and diseases, highlights the importance for developing crops which are able to withstand various environmental challenges simultaneously. In the present review we have provided evidence that enhancing the content of various low molecular weight antioxidants, isoprenoids, flavonoids, ascorbate, or glutathione increases the sustainability of higher plants under those factors, which are accompanied by the elevated ROS level.

The major knowledge presented in the literature about the role of enzymes in the synthesis of non-enzymatic antioxidants, as well as the characteristics of plant transformants with increased content of these antioxidants, was obtained using classical transgenes. The main aim of the present review was to summarize the information on existing techniques of plant engineering that have led to successful increases in non-enzymatic antioxidant production in order to identify the most promising future strategies. This review describes plenty of the examples of experimental works dedicated to overexpression of genes of the antioxidant’s biosynthesis. The review highlights that the most relevant strategy is to create overexpression of the genes, encoding the enzymes of the final stages of antioxidant biosynthesis. The attempts for overexpression of the genes of initial stages led to a rapid depletion of the common precursor for the biosynthesis of these antioxidants and to a decrease in their content and, as a consequence, to creation of stress-sensitive plants.

The described strategies for creating mutants by regulating the intensity of TF functioning are of importance, since the creation of such mutants can lead to intensified expression of a number of antioxidants. In this case, both overexpression of TF-activators and Crispr/Cas9 knockout of TF-repressors were successfully used [163,164]. When choosing tools for editing genes involved in the biosynthesis of antioxidants, it is worth paying attention to the characteristics of plant transformation and the target organs for increased antioxidant production. The widely used 35S promoter of cauliflower mosaic virus does not always lead to the desired effect, for example, when increasing the level of antioxidants in seeds. In such cases it is more relevant to use a seed-specific promoter. In addition to increasing the synthesis of the antioxidants itself, the strategy of parallel intensification of the production of corresponding chaperone proteins and storage sites for these substances is preferable, such as chromoplasts for carotenoids or plastoglobules for PQ and plastochromanol storage.

The current actively developing techniques of creating mutants using various genome editing methods significantly expands the capabilities of researchers. These techniques allow both the gene sequences and regulatory elements to be inserted into a certain given region of the plant genome. In addition, it makes it possible to introduce mutations into one or several target genes with an accuracy of one nucleotide. The genome editing methods are based on the manipulations of the nucleotide sequences in a strictly specified location and with a minimum number of off-target changes in the genome, and are supposed to be environmentally friendly. Nevertheless, transgenesis and genome editing are not mutually exclusive, but can complement each other. However, considering the above information concerning gene editing, it seems likely that the developing dCAs9 technique will displace the classical transgenesis for the creation of overexpressing mutants.

Overall, we have considered that the most prospective approach is the complex multi-strategy engineering, which, in addition to all of the above, also takes into account the fact that antioxidants are the precursors of some hormones and other metabolites. In this case, expression cassettes can be designed in such a way that some genes are overexpressed, while others are CRISPR/Cas9 knocked out or contain genes for RNA silencing. This was done, for example, in [146], where editing was carried out in relation to carotenoid synthesis genes, taking into account the possibility that carotenoid overproduction is able to delay seed germination [335], since carotenoids serve as precursors for ABA synthesis.

In addition to the versatility of such plants in terms of increased resistance to oxidative stress conditions, they also exhibit specific characteristics. This is a consequence of the fact that the non-enzymatic antioxidants discussed in this review perform other protective and signaling functions in plants, in addition to antioxidant functions. We have here described that creating plants with increased flavonoid content appears to be an effective means of combating biotic stresses, especially insect pests, since some of the flavonoids are toxic for herbivorous insects. Increasing glutathione content in plants may be an effective approach for phytoremediation of soils contaminated with heavy metals. Enhancing the content of ascorbic acid and of isoprenoids including UQ, PQ, and carotenoids improves the nutritional value of plants and increases the shelf life of fruits and seeds. Moreover, it is possible that under photoinhibitory conditions, the replacement of oxidized PQ derivatives in the thylakoid membrane by PQ molecules from plastoglobules can be facilitated in plants with increased PQ content. An increase in the carotenoid content, particularly of xanthophylls, is also important for plants, owing to their participation in the dissipation of excess energy into heat. This suggests that the creation of mutant plants with enhanced biosynthesis of antioxidants can be an effective strategy to increase the acclimatory potential of plants not only by reducing the level of ROS, but also by using the other defined internal potential reserves of plants.

As described in this review, the main pool of studies is devoted to the creation of plants with increased production of tocopherols, flavonoids, carotenoids, and ascorbate, while only a few articles have been published on the overexpression of PQ or UQ biosynthesis. These quinones are able to freely diffuse along the membranes, increasing the probability of their encountering ROS, which are produced by the photosynthetic and respiratory electron transport chain components. It can be predicted that their chemical and physical properties are the appropriate basis for manipulating the level of these components to improve plant sustainability.

Moreover, employing strategies for enhancement of biosynthesis of several antioxidants can lead to the development of plants with novel traits, addressing multiple challenges at once: enhancing plant resistance, elevating the nutritional value of crops, and aiding in the remediation of polluted soils.

## Figures and Tables

**Table 1:** *Arabidopsis thaliana* genes encoding the key enzymes involved in synthesis of isoprenoid antioxidants.

Genes	Enzyme and Its Alternative Names in Arabidopsis		Functions
*At5g16440*	IPPI, isopentenyl diphosphate isomerases		Enzymes with dimethyl allyl diphosphate isomerase activity. It is involved in the biosynthesis of IPP, isopentenyl diphosphate. IPP is a subject for further condensation reactions to form intermediates in the synthesis of plastidic and mitochondrial isoprenoids (carotenoids, tocopherols, PQ, plastochromanol, UQ)
*At3g02780*			
*At4g19010*	AT4G 19010	Peroxisomal 4-coumarate CoA ligases	4-HBA (hydroxybezoic acid) biosynthesis from phenylalanine in peroxisomes for further UQ biosynthesis [16,17]
*At5g38120*	4CL8		
*At5g47770*	FPPS1	Farnesyl diphosphate synthases	Isoprenoid farnesyl diphosphate (FPP) biosynthesis for further UQ biosynthesis [18,19]
*At4g17190*	FPPS2		
*At2g34630*	CoQ1, SPS3, solanesyl diphosphate synthase		Isoprene polymerization for further UQ biosynthesis [20]
*At4g23660*	Coq2, PPT1, 4-hydroxybenzoate polyprenyl diphosphate transferase		Rate-limiting enzyme in UQ biosynthesis. Catalysis of benzoquinone ring of 4-HB condensation with polyisoprenoid side chain of polyprenyl pyrophosphate to form 3-polyprenyl-4-hydroxybenzoate [21]
*At3g24200*	Coq6	Flavin-dependent monooxygenases	Aromatic hydroxylation of C-H in different positions in UQ biosynthesis
*At1g24340*	CoqF		
*At2g30920*	Coq3	S-adenosyl-l-methionine (SAM)-dependent methyl transferases	
*At5g57300*	Coq5		
*At2g03690*	Coq4		Presumably a scaffold protein, which is responsible for organization of UQ biosynthetic complex [22]
*At5g17230*	PSY, phytoene synthase		Condensation of two molecules of GGDP to produce phytoene for further carotenoid biosynthesis
*At4g14210*	PDS, phytoene desaturase		Desaturation of phytoene to ζ-carotene by introduction of four double bonds into phytoene for further carotenoid biosynthesis
*At3g04870*	ZDS, ζ-carotene desaturase		Reduction of ζ-carotene to lycopene by introduction of four double bonds for further carotenoid biosynthesis
*At1g06820*	CRTISO, carotenoid isomerase		Catalyzes cis–trans isomerization of poly-cis-carotenoids to all-trans-lycopene. Together with PDS and ZDS, CRTiso is required to complete the synthesis of lycopene from phytoene for further carotenoid biosynthesis [23,24]
*At3g10230*	βLCY1, β-carotene cyclase, ATLCY, LYC, Lycopene cyclase		Introduction of a ring at both ends of symmetrical lycopene to form the bicyclic β-carotene [25]
*At5g57030*	εLCY, ε-carotene cyclase		Required to form lutein [26]
*At4g25700*	β-OHase1	β-carotene hydroxylases	Conversion of beta-carotene to zeaxanthin via cryptoxanthin [27]
*At5g52570*	β-OHase2		
*At3g53130*	εOHase, ε-carotene hydroxylase		Involved in epsilon ring hydroxylation to carotene for lutein biosynthesis [28]
*At5g67030*	ZE, zeaxanthin epoxidase		Introduction of epoxide groups into both rings of zeaxanthin to form violaxanthin [29]
*At1g08550*	VDE,		De-epoxidation of violaxanthin to zeaxanthin [30]
*At1g06570*	HPPD, 4-hydroxyphenylpyruvate dioxygenase (α-ketoisocaproate dioxygenase, KIC dioxygenase)		Homogentisate (HGA) synthesis from hydroxyphenylpyruvate for further biosynthesis of PQ, plastochromanol, and tocopherols [31]
*At5g04490*	VTE5, phytol kinase		Phosphorylation of free phytol for further biosynthesis of tocopherols [32]
*At1g78620*	VTE6, phytyl-phosphate kinase		A key enzyme for phytol phosphorylation for further biosynthesis of tocopherols and phylloquinone [33]
*At1g78510*	SPS1	Solanesyl diphosphate synthases	Solanesyl diphosphate condensation from geranylgeranyl diphosphate (GGDP) and isopentenyl phosphate (IPP) for further biosynthesis of PQ and plastochromanol [34,35,36]
*At1g17050*	SPS2		
*At5g09820*	FBN5-B	Fibrillins	Specifically interacted with solanesyl SPS1 and SPS2
*At3g11945*	HST, homogentisate solanesyl diphosphate transferase		Condensation of homogentisate (HGA) with solanesyl diphosphate with formation of methyl-solanesyl-benzoquinone (MSBQ) for further biosynthesis of PQ and plastochromanol
*At2g18950*	HPT, homogentisate phytyl transferase (VTE2)		Catalysis of condensation of HGA and Phytyl-DP to form dimethyl-phytyl-benzoquinone (MPBQ) for further biosynthesis of tocopherols
*At3g63410*	VTE3 2-methyl-6-phytyl-1,4-benzoquinol methyltransferase		Methyl-solanesyl-benzoquinone (MSBQ) conversion to PQ and methyl-phytyl-benzoquinone (MPBQ) conversion to DMPBQ for further biosynthesis of tocopherols [37]
*At4g32770*	VTE1, tocopherol cyclase		Plastochromanol-8 synthesis from PQH_2_; α-tocopherol biosynthesis from γ-tocopherol [37]
*At1g64970*	VTE4, G-TMT, γ-tocopherol methyltransferase		Conversion of δ- and γ-tocopherols (and tocotrienols) to β- and α-tocopherols [37]

**Table 2:** Engineering plants through CRISPR/Cas9 editing of the genes involved in synthesis of antioxidants of non-enzymatic nature.

Proteins	Species	Target Genes	Anti-Oxidants	Editing Type	Result
Kaempferol 3-O-rhamnosyltransferase and kaempferol 3-O-glucosyltransferase	*A. thaliana*	*At1g30530*, *At5g17050*	UQ	Knockout as a result of deletion and insertion	UQ content in the double knockout represented 160% of wild-type level [147]
PSY, phytoene synthase	*Oryza sativa*	*ZmPsy*	Carotenoids	Marker-free targeted insertion at pre-determined plant genomic safe harbors (knockin *Erwinia uredovora carotenoid desaturase* (*SSU-crtI*) and *maize phytoene synthase* (*ZmPsy*) both driven by the endosperm-specific glutelin promoter)	High level of β-carotene in the endosperm [145]
SlCYC-B, lycopene-β-cyclase; SlDDB1, DNA damage UV binding protein 1; SlDET1, de-etiolated1	*Solanum* *lycopersycum*	*DNA damage SlCYC-B*, *SlDDB1*, *SlDET1*,	Carotenoids	Target activation-induced cytidine deaminase base-editing technology, substitution of a cytidine with a thymine	Variations in carotenoid accumulation with an additive effect for each single mutation [138,139]
*LCY-E*, lycopene ε-cyclase; *Blc*, beta-lycopene cyclase; *LCY-B1*, lycopene β-cyclase 1; *LCY-B2*, lycopene β-cyclase 2; *SGR1*, Stay-green 1	*S. lycopersycum*	*DQ100158* (*SGR1*), *EU533951* (*LCY-E*), *XM_010313794* (*Blc*), *EF650013* (*LCY-B1*), *AF254793* (*LCY-B2*)	Carotenoids	Knockout as a result of deletions, insertion, substitution	Lycopene content in tomato fruit was increased about 5.1-fold [132]
*LCYE*, lycopene ε-cyclase	*O. sativa* (rice calli)	*LcyE*		Gene replacement using HDR, substitution H523L	Orange-colored line, total carotenoid content was 6.8–9.6 times higher than that of wild-type calli, increased tolerance to salt stress [135]
	*Nicotiana tabacum*	*Ntε-LCY1*, *Ntε-LCY2*		Knockout as a result of deletions, insertion, substitution	Increase in the total carotenoid and chlorophyll contents, photosynthetic efficiency, and levels of the stress response [137]
	*Musa sapientum* (*banana*)	*GN-LCYε*		Knockout as a result of indels	Accumulation of β-carotene content up to 6-fold; absence or a drastic reduction in the levels of lutein and α-carotene [134]
EIL2, Ethylene-Insensitive 3/ Ethylene-Insensitive 3-Likes	*S. lycopersycum*	*EIL2*	Carotenoids, Ascorbate	Knockout as a result of insertion	Yellow, orange fruits; 1.62-fold increase of ascorbate content via both the L-galactose and myoinositol pathways [67]
PDS, phytoene desaturases	*Malus domestica* (apple)	*LC10183* (*PDS*)	Carotenoids	Knockout as a result of deletions, insertion	Albino phenotypes of regenerated plantlets [50]
	*Fragaria* sp.	*PDS*		Knockout as a result of deletions	Albino regenerants [51]
	*Daucus carota* (Orange carrot ‘Kurodagosun’, ‘Deep purple’ carrot)	*XM_017385289.1* (*DcPDS* and *DcMYB113-like genes*)		Knockout as a result of deletions, insertion, substitution	Albino plants and purple color depigmented plants [54]
	*Dioscorea rotundata*	*DrPDS*		Knockout as a result of deletions, insertion	Phenotypes of variegated to complete albinism [52]
	*Allium cepa* L.	*AcPDS*		Knockout as a result of deletions, indels	Regenerated shoots exhibited three distinct phenotypes: albino, chimeric, and pale green [53]
CCDs, carotenoid cleavage dioxygenases	*Musa sapientum* (banana)	*CCDs*	Carotenoids	Knockout as a result of deletions	Higher fold β-carotene accumulation in non-green tissue (roots) than in green tissue (leaf) [144]
β-OHase2, β-carotene hydroxylase	*A. thaliana*	*At5g52570* (*BCH2*)	Xanthophylls	Knockout as a result of deletions	Prevention of the negative effects of carotenoid overproduction on seed germination [146]
DnaJ, cysteine-rich zinc-binding domain	*O. sativa* (rice calli)	*Orange gene* (*OsOr*)	Chromoplast formation	Knockout as a result of deletions	Orange-colored line accumulated more lutein, *β*-carotene, and two *β*-carotene isomers; increased tolerance to salt stress [142]
F3H, flavanone 3-hydroxylases	*D. carota* (Carrot calli, purple-colored)	*F3H*	Dihydro-flavonols, leucoantho-cyanidins, pro-anthocyanidins, anthocyanidins, anthocyanins	Knockout as a result of deletions	Blockage of the anthocyanin biosynthesis, discoloration of calli [148]
F3′H, flavanone 3′-hydroxylase	*Oryza sativa* L. (black rice)	*Os10g0320100* (*OsF3′H*)	Flavan-3-oles	Knockout as a result of deletions, insertions	Ocher seeds, much lower anthocyanin content [149]
	*Euphorbia pulcherrima*	*F3′H*			Increased ratio of pelargonidin to cyanidin, bright color changed from vivid red to vivid reddish orange [150]
DFR, *dihydroflavonol 4-reductase*	*Zea mays*	*GRMZM2G026930* (*a1*), *MZM2G013726* (*a4*)	Leucoantho-cyanidins, pro-anthocyanidins, anthocyanidins, anthocyanins	Knockout as a result of deletions, insertions	Blockage of the anthocyanin biosynthesis [151]
	*S. lycopersycum*	Solyc02g085020 (*DFR*)			Blockage of the anthocyanin biosynthesis, hypocotyls and callus were green [152,153]
	*Oryza sativa* L. (black rice)	*Os01g0633500* (*OsDFR*)			Much lower anthocyanin content, ocher seeds [149]
	*Ipomoea nil*	*AB006793* (*InDFR-B*)			Anthocyanin-less white flowers [154]
	*S. lycopersycum*	*DFR*			Green hypocotyl due to defective anthocyanin accumulation [153]
LDOX, leucoanthocyanidin dioxygenase	*Oryza sativa* L. (black rice)	*Os01g0372500* (*OsLDOX*)	Prontho-cyanidins, anthocyanidins, anthocyanins	Knockout as a result of deletions and insertions	Brown seeds, much lower total anthocyanin content [149]
UGTs, UDP-glucosyltransferases	*A. thaliana*	*UGT79B2* (*At4g27560*), *UGT79B3*, (*At4g27570*)	Modulating anthocyanin biosynthesis and abiotic stress tolerance	Knockout as a result of deletions and insertions	Reduced levels of flavonoids and increased susceptibility to abiotic stress [155]
*Gt5GT*, anthocyanin 5-*O*-glucosyltransferase; *Gt3′GT*, anthocyanin 3′-*O*-glucosyltransferase; *Gt5/3′AT*, anthocyanin 5/3′-aromatic acyltransferase	*Gentian* cv. Albireo (*Gentiana-triflora* × *Gentianascabra*)	*Gt*5*GT*, *Gt3*′*GT*, *Gt*5*/3*′*AT*	Anthocyanin biosynthesis	Knockout as a result of deletions and insertions	Transformants produced pale red-violet, dull pink, and pale mauve flowers [156]
PAP1, production of anthocyanin pigment 1 (MYB transcription factor (TF))	*A. thaliana*	*AT1G56650* (*PAP1*)	Flavonoids	CRISPR/Cas9 activation system with the p65-HSF activators to increase endogenous transcriptional levels	Purple pigmentation of the leaves under a high light [157]
*ANT1*, anthocyanin mutant 1 (Myb TFs)	*S. lycopersicum*	*ANT1*	Flavonoids	Gene targeting upstream of the *ANT1* gene	Overexpression and ectopic accumulation of pigments in tomato tissues [158]
				CRISPR/LbCpf1-based HDR, gene targeting upstream of the *ANT1* gene	Tomato purple phenotype with salinity tolerance [159]
SlAN2-like, (R2R3-MYB TFs)		*Solyc10g086290* (*SlAN2-like*)		Knockout as a result of deletion	Lower accumulation of anthocyanins, downregulation of multiple anthocyanin-related genes [160]
SlAN2 (R2R3-MYB TFs)		*SlAN2*		Knockout as a result of deletion and substitution	Flavonoid content and the relative expression levels of several anthocyanin-related genes in vegetative tissues were significantly lower [161]
DcPDS and DcMYB113-like (R2R3-MYB TFs)	*D. carota* (‘Deep Purple’)	*DcPDS*, *DcMYB113-like*		Knockout as a result of deletions	Regenerated albino shoots [54]
PtrMYB57 (R2R3-MYB TFs)	*Populus tomentosa* Carr	*PtrMYB57*	Anthocyanin and proanthocyanidin	Knockout as a result of deletions	High anthocyanin and proanthocyanidin phenotype [162]
FtMYB45 (R2R3-MYB TFs)	*Fagopyrum tataricum*	*FtMYB45*	Flavonoids	Knockout as a result of deletions and insertion	Content of rutin, catechin, and other flavonoids was increased in hairy root mutants [163]
bZIP (basic region/leucine zipper TFs)	*Vitis vinifera*	*VvbZIP36*	Flavonoids	Knockout as a result of deletions and insertion	Accumulation of metabolites (naringenin chalcone, naringenin, dihydroflavonols, and cyanidin-3-O-glucoside); synthesis of stilbenes (α-viniferin), lignans, and some flavonols (including quercetin-3-O-rhamnoside, kaempferol-3-O-rhamnoside and kaempferol-7-O-rhamnoside) was significantly inhibited [164].
TTG1, Transparent Testa Glabra1 (MYB-bHLH-WD40 TFs)	*A. thaliana*	*TTG1*	Flavonoids	Knockout as a result of deletion	Mutants produce pale seeds and lack trichomes [165]
	*O. sativa* L.	*OsTTG1*			Decreased falvonoid accumulation in various rice organs [166]
TT, transparent testa (bHLH TFs)	*Brassica napus*	*BnTT8*	Proanthocyanidin	Knockout as a result of deletion and insertion	Yellow-seeded phenotype, seeds with elevated seed oil and protein content, and altered fatty acid composition [167,168]
	*N. tabacum* L.	*NtAn1a*, *NtAn1b*			
		uORF*GGP1*		Single nucleotide transversion from C to T in the 5′ UTR of the Solyc06g073320 sequence, leading to a change in the predicted amino acid sequence from serine to phenylalanine	Increased ascorbate content (two- to five-fold higher), male sterility [169]
GST, Glutathione S-transferase	*S. lycopersycum*	SlGSTAA	Quenching of the toxic compounds together with glutathione	Knockout as a result of deletions	Green hypocotyl owing to anthocyanin deficiency [170,171]
	Gentian cv. Albireo (*G. triflora* × *G. scabra*)	*GST*		Knockout as a result of deletions	Decreased anthocyanin accumulation in flower petals [156]
	*F. vesca*	*RAP*, *Reduced Anthocyanins in Petioles*		Knockout as a result of deletions, insertion	Green stem and white-fruited phenotype [172]
Phosphorylase, GGP	*Lactuca sativa*	uORF_AtVTC2_*LsGGP1* and *LsGGP2* (homologs of *AtVTC2*)	Ascorbate	Knockout as a result of deletions and indels	Increased ascorbate content by ~150% and oxidation stress tolerance [173]
	*S. lycopersicum*	uORF_AtVTC2_*LsGGP2* (homologs of *AtVTC2*)		Knockout, deletions, indels	Increased ascorbate content [132]

**Table 3:** *Arabidopsis thaliana* genes encoding the key enzymes involved in synthesis of flavonoids.

Genes	Enzyme and Its Alternative Names in Arabidopsis	Functions
*At2g37040*	PAL, phenylalanine ammonia lyase	The deamination of phenylalanine to trans-cinnamic acid [178,179]
*At3g53260*		
*At2g30490*	C4H, Cinnamic acid 4-hydroxylase	The hydroxylation of trans-cinnamic acid [180,181]
*At1g65060*	4CL, 4-coumarate CoA ligase	Coumaric acid conversion to coumaroyl-CoA, which is the last step of phenylpropanoid pathway [182]
*At5g13930*	CHS, chalcone synthase (ATCHS, Transparent Testa 4, TT4)	The condensation of activated coumaric acid with three molecules of activated malonic acid in the form of malonyl-CoA to the formation of naringenin-chalcone. A key enzyme involved in the biosynthesis of flavonoids [183]
*At3g55120*	CHI, chalcone isomerase (A11, ATCHI, Chalcone flavanone isomerase, Transparent Testa 5, TT5)	Catalysis of the conversion of chalcones into flavanones [183]. *At3g55120* is co-expressed with CHS encoding gene [60]
*At3g51240*	F3H, flavanone 3-hydroxylase	Encodes flavanone 3-hydroxylase that is coordinately expressed with CHSs and CHIs and involved in flavonoid biosynthesis [184]
*At5g07990*	F3′H, flavanone 3′-hydroxylase (CYP75B1, Cytochrome P450 75B1, D501, Transparent Testa 7, TT7)	Hydroxylation of 3′-position of B-ring of flavonoids with catalysis of dihydroquercetin and quercetin formation from dihydrokaempferol and kaempferol, respectively [184]
*At5g24530*	FNS, flavone synthase (AtDMR6, Downy Mildew Resistant6)	The conversion of the flavanones into flavones. This class is also shown to comprise soluble Fe^2+^/2-oxoglutarate-dependent dioxygenases, which are oxygen- and NADPH-dependent cytochrome P450 membrane-bound monooxygenases [185]
*At5g08640*	FLS, flavonol synthase (ATFLS1)	Encodes a flavonol synthase that catalyzes formation of flavonols from dihydroflavonols. Co-expressed with CHI and CHS (qRT-PCR)
*At5g63590*		
*At5g42800*	DFR, dihydroflavonol 4-reductase	The reduction of the 4-keto group of dihydroflavonol to the corresponding leucoanthocyanidin. Synthesis of phlobaphenes from flavan-4-oles in *Zea mays* [186]
*At1g61720*	ANR, anthocyanidin reductase	Synthesis of proanthocyanidins (condensed tannins) from leukoanthocyanidins and anthocyanidins [13]
*At4g22880*	ANS, anthocyanidin synthase (LDOX, Leucoanthocyanidin dioxygenase)	Convertion leucoanthocyanidins to anthocyanins [187]
*At5g17050*	FGT, flavonoid glycosyltransferases	Glycosylation of anthocyanidins to anthocyanins [188,189,190,191]
*At1g30530*		
*At5g17030*		
*At2g36790*		
*At1g06000*		
*At4g14090*		
*At5g54060*		
*At2g47460*	MYB domain protein 12, MYB12, ATMYB12, PFG1	Flavonol synthesis regulators. Strongly activate the promoters of CHS, F3H, FLS, and CHI [192]
*At3g62610*	AtMYB11, PFG2	
*At5g49330*	AtMYB111, PFG3	
*At2g46510*	bHLH17 (ABA-inducible bHLH-type transcription factor), AIB, ATAIB, JA-associated MYC2-like 1, JAM1	Positive regulator of flavonoid biosynthesis [193]

**Table 4:** *Arabidopsis thaliana* genes encoding the key enzymes involved in synthesis of glutathione and ascorbate.

Genes	Enzyme and Its Alternative Names in Arabidopsis		Functions
*At4g23100*	γ-glutamylcysteine synthetase, γ-ECS, ATECS1, ATGSH1, Cinnamyl Alcohol Dehydrogenase Homolog 2, Glutamate-Cysteine Ligase, GSH1, GSHA		Catalysis of the first, and rate-limiting, step of glutathione biosynthesis.
*At5g27380*	Glutathione Synthetase 2, ATGSH2, GSH2, GSHB		Binding γ-glutamylcysteine and glycine together to form glutathione
*At4g29130*	Hexokinase 1, HXK1, ATHXK1, GIN2		Hexose phosphorylation activity
*At2g19860*	Hexokinase 2, HXK2, ATHXK2		
*At1g47840*	Hexokinase 3, HXK3		
*At4g24620*	Phosphoglucose Isomerase, PGI, Glucose-6-phosphate isomerase		Transformation of d-glucose-6-phosphate into d-fructose-6-phosphate
*At3g02570 At1g67070*	Man-6-phosphate Isomerase, Phosphomannose isomerase, PMI		D-mannose-6-P formation from d-fructose-6-phosphate [264]
*At2g45790*	Phosphomannomutase, PMM		Transformation of D-mannose 6-phosphate into D-mannose 1-phosphate [265,266]
*At2g39770*	GDP-D-mannose pyrophosphorylase, GMP1, Vitamin C Defective 1, VTC1, Cytokinesis Defective 1, CYT1, Embryo Defective 101, EMB101, Sensitive To Ozone 1, SOZ1,		Guanosine monophosphate transfer from GTP to GDP-D-Mannose [254,267,268]
*At5g28840*	GDP-D-mannose 3′,5′ Epimerase, GME		The conversion of GDP-D-mannose to GDP-L-galactose. GME is also able to catalyze the 3′ epimerization of GDP-mannose, giving GDP-l-gulose, which is the precursor of a possible side-branch biosynthetic pathway (the gulose pathway) for vitamin C synthesis [255,269]. Plays a key role at the intersection of ascorbate and non-cellulosic cell-wall biosynthesis
*At5g55120*	VTC5	GDP-L-Galactose Phosphorylase, GGP	Encodes a novel protein involved in ascorbate biosynthesis, which has been shown to catalyze the transfer of GMP from GDP-galactose to a variety of hexose-1-phosphate acceptors [270]
*At4g26850*	VTC2		
*At3g02870*	L-Galactose 1-P-phosphatase, GPP, VTC4		Conversion of l-Galactose-1-phosphate into l-galactose [271,272,273]
*At3g07130*	Purple acid phosphatase with phytase activity, PAP15		
*At4g33670*	L-Galactose Dehydrogenase, GDH		Conversion of l-galactose into l-galactono-1,4-lactone [254]
*At3g47930*	L-Galactono 1,4-lactone Dehydrogenase, GLDH		Oxidation of L-galactono-1,4-lactone to Asc [267,274]
*At3g05620* *At5g04970* *At5g47500* *At5g61680*	Methyl Esterases		Conversion of Methyl-D-Galacturonate into D-Galacturonate in the D-Galacturonate pathway [275]
*At1g14520* *At4g26260*	Myo-Inositol Oxygenase, MIOX1		Convertion of Myo-inositol into L-Gulono-1,4-lactone Myo-inositol [257]
*At1g65770*	Ascorbic Acid Mannose Pathway Regulator 1, AMR1, ATFDA7, F-BOX/DUF295 ANCESTRAL 7		Regulation of the mannose/L-galactose ascorbic acid biosynthetic pathway in response to developmental and environmental factors [276]
*At3g23230*	Ethylene Response Factor 98, ERF98, AtERF98, Transcriptional Regulator of Defense Response 1, TDR1, TTDR1		Enhancement of the tolerance to salt through the transcriptional activation of ascorbic acid synthesis [277]

## Data Availability

The original research results presented in this paper have not been submitted to other journals, and were not previously published.
